# Efficacy of intravesical therapies on the prevention of recurrence and progression of non‐muscle‐invasive bladder cancer: A systematic review and network meta‐analysis

**DOI:** 10.1002/cam4.3513

**Published:** 2020-10-11

**Authors:** Jun‐Lin Lu, Qi‐Dong Xia, Ying‐Hong Lu, Zheng Liu, Peng Zhou, Heng‐Long Hu, Shao‐Gang Wang

**Affiliations:** ^1^ Department of Urology Tongji Hospital Tongji Medical College Huazhong University of Science and Technology Wuhan China; ^2^ Department of Epidemiology, School of Public Health Fudan University Shanghai China

## Abstract

Intravesical instillation therapy is the mainstay of prophylaxis of tumor recurrence and progression in non‐muscle‐invasive bladder cancer. However, there is no study evaluating the superiority of monotherapy. The aim of this study is to compare the efficacy of preventing recurrence and progression of intravesical monotherapies via network meta‐analysis (NMA) of randomized controlled trials. Database searches were conducted on Embase, Ovid Medline, Web of Science, ScienceDirect, Cochrane Library, and ClinicalTrials.com from the time of establishment to February 6, 2020. The monotherapies included Bacille Calmette‐Guérin (BCG), mitomycin C (MMC), interferon (IFN), adriamycin, epirubicin, gemcitabine (GEM), and thiotepa (THP). A Bayesian consistency network model was generated under a random‐effects model. The superiority of therapy was identified based on the surface under the cumulative ranking curve (SUCRA). Fifty‐seven studies with 12462 patients are included. NMA shows that GEM (SUCRA = 0.92), BCG (SUCRA = 0.82), and IFN (SUCRA = 0.78) are the top three effective drugs to reduce recurrence. GEM (SUCRA = 0.87) is the most effective therapy to prevent progress, followed by BCG, MMC, THP, and IFN with similar efficacy. Subgroup analysis of pairwise meta‐analysis and NMA was performed on publication year, trial initiation year, study origin, center involvement, sample size, drug schedule, tumor characteristics, and trial quality to address confounding factors, which suggests the robustness of the results with stable effect sizes. Network meta‐regression also indicates consistent rank by analyzing year, sample size, and quality. Compared with BCG, GEM is also a promising therapy with favorable efficacy to reduce tumor recurrence and progression. IFN and MMC could be alternative therapies for BCG with slightly inferior efficacy in recurrence prevention and similar efficacy in progression prevention. However, the results of this study should be treated with caution since most of the included studies are of moderate to high risk of bias.

## Graphical abstract

The meta‐analysis of randomized controlled trials evaluates the efficacy of intravesical monotherapies in preventing tumor recurrence and progression in patients with non‐muscle‐invasive bladder cancer. The results identify potential alternative therapies for BCG, such as gemcitabine, interferon, and mitomycin C, which can be considered the optimal second‐line therapy.

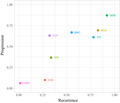

## 1 INTRODUCTION

Bladder cancer is the 9th most common malignancy globally and its cancer‐related mortality rate is the 13th highest.^1^ Approximately 75% patients are initially diagnosed as non‐muscle‐invasive bladder cancer (NMIBC).^2^ NMIBC is characterized by high recurrence and progression rates, and the probability of recurrence ranges from 15% to 61% at 1 year and 31% to 78% at 5 years; furthermore, 1%–45% of NMIBCs will progress into muscle‐invasive bladder cancer (MIBC) at 5 years.^3^ Patients with MIBC have much poorer survival rates (29%–57% at 5 years) than NMIBC.^4^, ^5^ Radical cystectomy, the standard treatment for MIBC, negatively impacts the quality of life, emotional function, and cognitive function of patients.^6^ Therefore, it is essential to investigate strategies for reducing the recurrence and progression rates of NMIBC.

Intravesical instillation therapies administered after transurethral resection of bladder tumor (TURBT) have demonstrated a capacity to prevent the recurrence of NMIBC. Among the therapies, Bacille Calmette‐Guérin (BCG) is considered the most effective treatment.^7^ Scheduled BCG instillation can achieve a 68.1% initial complete response rate and a 46.7% disease‐free rate based on a median follow‐up of 3.6 years.^8^ However, the majority of patients will suffer toxicity such as cystitis and flu‐like symptoms, and some patients will even develop systemic tuberculosis infections.^9^, ^10^ In addition, currently, the BCG strain is in short supply globally since two major producers cited disruptions in BCG supply due to manufacturing challenges.^11^ Therefore, a comprehensive evaluation of the efficacy of other intravesical therapies and identifying alternative therapies is vital.

Network meta‐analysis (NMA) is an extended qualitative synthesis of traditional pairwise meta‐analysis (PMA). It integrates direct effects within trials with indirect effects between trials based on Bayesian theory, with the aim of estimating the network efficacy based on therapeutic rank.^12^ Previous NMA did not evaluate intravesical therapy as monotherapy.^13^, ^14^ Besides, the previous NMAs used odd ratio instead of time‐dependent hazard ratio to obtain a mixed effect sizes.^13^, ^14^ Therefore, the present NMA could reveal the optimal intravesical therapy in addition to the potential second‐line treatment through comparisons of the efficacy of common intravesical drugs: BCG, mitomycin C (MMC), interferon (IFN), adriamycin (ADM) or doxorubicin, epirubicin (EPI), gemcitabine (GEM), and thiotepa (THP).

## 2 METHODS

The present NMA was performed in accordance with the Preferred Reporting Items for Systematic Reviews and Meta‐Analyses (PRISMA) guideline for NMA.^15^ The PRISMA NMA checklist is accessible and can be used to evaluate the compliance of the present meta‐analysis (File S1).

### 2.1 Search strategy

A database search was conducted in February 2020. Two investigators (J. L. and Q. X.) searched six electronic databases (Embase, Ovid Medline, Web of Science, ScienceDirect, Cochrane Library, and clinicaltrial.com) between the time of launching of the database and February 6, 2020. Seven intravesical therapies were included in the present meta‐analysis, namely, BCG, MMC, IFN, ADM, EPI, GEM, and THP. The detailed search procedure is described in File S2. Google Scholar was used to retrieve grey literature. The references in relevant meta‐analyses were also reviewed.

### 2.2 Selection criteria

A study would be included in the meta‐analysis if it satisfies the predefined (patient, interventions, comparators, outcomes, and study design) PICOS criteria:

- Patient: pathologically diagnosed as non‐muscle‐invasive or superficial or Ta/T1 with/without Tis bladder cancer. There were no restrictions to age, sex, race, pathological grade, and previous intravesical therapy.
- Interventions: monotherapy from the seven selected therapies after TURBT. The number of instillations was greater than two. Combined therapy and immediate instillation were excluded.
- Comparators: another intravesical therapy after TURBT or only TURBT. The number of instillations was greater than two.
- Outcomes: recurrence or progression (to T2 or greater) of bladder cancer confirmed by pathologists. Studies were eligible if they reported at least one of the two outcomes.
- Study design: two or multiple arms randomized controlled trial.

All included publications were restricted to peer‐reviewed studies in the English language. If one trial was reported in more than one publication, the most recent or informative publication was included.

### 2.3 Data extraction and quality assessment

Two reviewers (Q. X. and J. L.) extracted the data using standardized forms. The data were as follows: name of the first author, publication year, trial initiation year, study origin, study center, study duration, the number of eligible patients, the number of analyzed patients, baseline age, sex, tumor characteristics (primary/recurrent, risk of recurrence, pT stage, grade, and carcinoma in situ), intervention (description, BCG strains, dosage, and schedule), and outcome. The outcome of each study was evaluated based on computed log‐hazard ratios (logHR) and their standard errors.^16^, ^17^ Log hazards and corresponding standard errors of each arm were calculated in multi‐arm trials.^18^ We stratified intravesical instillation schedule into induction schedule and maintenance schedule. Instillations less than 8 times or continuous weekly (or twice weekly) instillation were considered induction schedules. Instillations greater than 8 as well as monthly (or twice monthly) instillations after an induction schedule were recorded as maintenance schedules. The two reviewers independently conducted critical appraisals using the version 2 of the Cochrane tool for risk‐of‐bias tool for randomized trials (RoB2).^19^ The revised RoB2 tool assesses five distinct domains: the randomization process, deviations from intended interventions, missing outcome data, measurement of the outcome, and selection of the reported result. Disagreements were resolved by discussion. We assigned 0 to 2 scores to “high risk of bias”, “some concerns”, and “low risk of concerns” in each domain. A total score ranging from 0 to 10 was then obtained for each study. The quality of evidence was assessed using the Grading of Recommendation, Assessment, Development, and Evaluation (GRADE) approach for NMA.^20^

### 2.4 Statistical analysis

We performed PMA with “meta” package in R v4.0.0. We calculated the pooled effect size as hazard ratio (HR) with 95% confidence interval using a random‐effects model. The Cochrane *Q* test, *I* ^2^, and *τ* ^2^ were used to assess heterogeneity. Subgroup and sensitivity analysis was conducted when more than two random‐controlled trials (RCTs) were included in comparisons. Subgroups were obtained based on study and patient characteristics: publication year, trial initiation year, study origin, study center, sample size, therapy schedule, study quality, adverse effect reported, and tumor characteristics. If comparisons included at least ten RCTs, meta‐regression and publication bias were tested using Egger's test.

The NMA was based on the Bayesian random‐effects model and was conducted with the “gemtc” and “rjags” packages in R v4.0.0. The NMA was based on the consistency model and computed using four Markov chains, each with 5000 burn‐in iterations and 20000 Markov chain Monte Carlo (MCMC) transitions. The consistency of the model was evaluated using the node‐split method. The pooled effect sizes were acquired as HR and 95% credible intervals. The robustness of the NMA results was tested using subgroup analysis and meta‐regression. The subgroup analyses were run with the same MCMC parameters (chain number, burn‐in iterations, and transitions). The relative rankings of seven therapies were estimated based on the surface under the cumulative ranking curve (SUCRA).^21^ The SUCRA values were obtained from the distribution of the ranking probabilities. The difference was considered statistically significant when the *P*‐value was less than 0.05.

## 3 RESULTS

### 3.1 Search results and study characteristics

**FIGURE 1:**
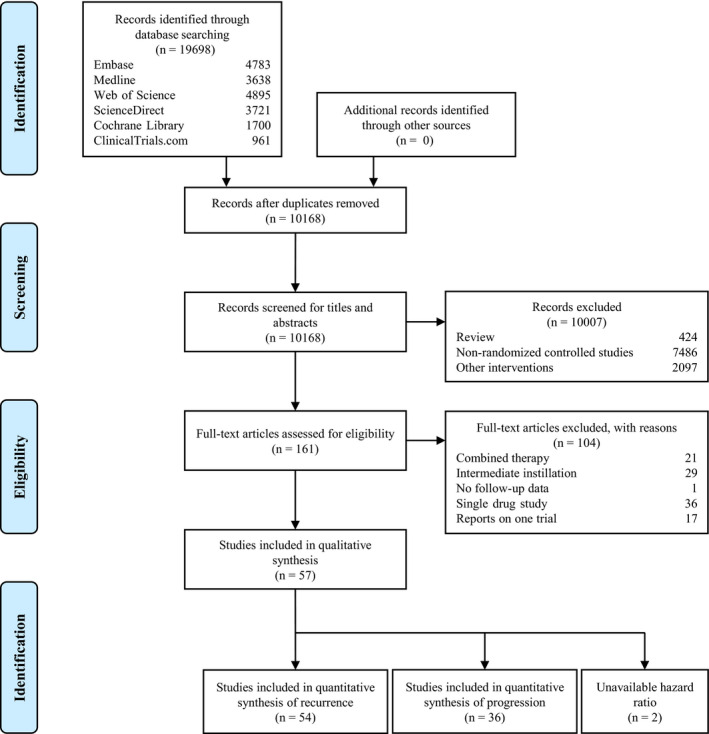
Flowchart of study selection

Of 19,689 records initially identified, 161 RCTs were assessed by full‐text review (Figure 1). The detailed reasons for excluding 104 RCTs are summarized in File S3. Fifty‐seven trials are finally included in the analyses. The quantitative analysis is not conducted on two trials since their HRs were unavailable.^22^, ^23^ The meta‐analysis is based on 54 trials of recurrence and 36 trials of progression. The studies are published between 1982 and 2013. Eleven trials are conducted in Japan, seven trials are carried out in Greece, whereas six trials are conducted in Italy. The study and patient characteristics are summarized in Table S1 (File S4). A total of 132 arms with 12462 enrolled participants are included in the quantitative synthesis. Among the 132 arms, 29 arms receive BCG, 26 arms receive MMC, 20 arms receive ADM, 14 arms receive EPI, 11 arms receive IFN (including IFNα‐2a, IFNα‐2b, IFN‐α, and IFN γ‐1b), 4 arms receive GEM, 4 arms receive THP, whereas 24 arms do not receive any treatment after TURBT. The interventions and outcome effect sizes are summarized in Table S2 (File S4).

The quality assessment using RoB2 is illustrated in Figure S1 and summarized in Figure S2 (File S5). Twenty‐seven studies exhibit high risk of bias, whereas twostudies have low bias risk. In addition, 26 other trials have some risk of bias concerns. Unclear risk mainly results from unspecified randomization process (61.8%) and deviations from intended interventions (87.3%). The “missing outcome data” domain contains 41.8% of the studies with high risk of bias.

### 3.2 Pairwise meta‐analysis results

**FIGURE 2:**
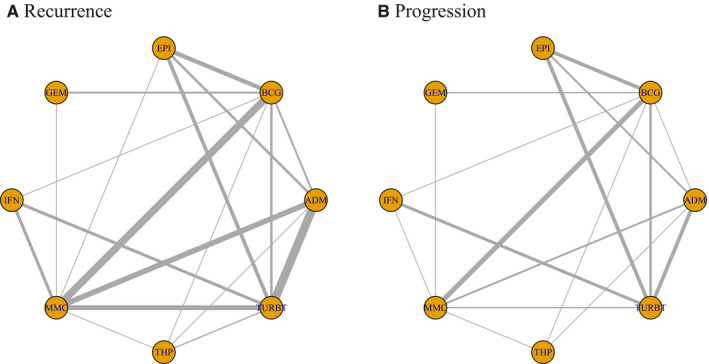
Network diagrams of direct comparisons on recurrence (A) and progression (B)

**FIGURE 3:**
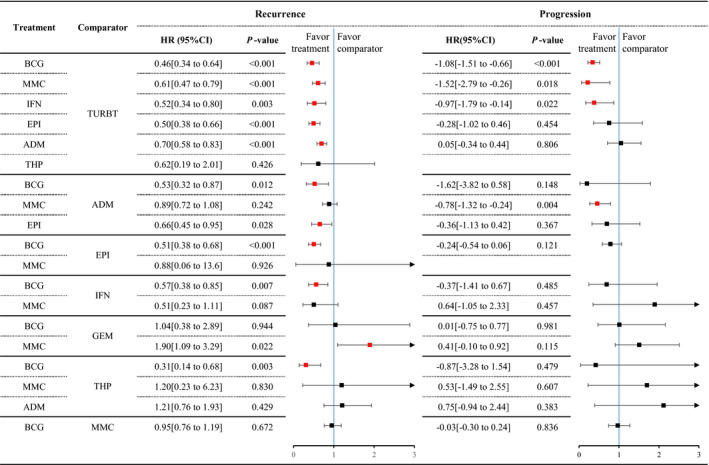
Forest plot of pairwise meta‐analysis. Red point of effect size indicates that the difference of the comparison is significant

Figure 2 presents the network diagrams of direct comparisons. Effect sizes estimated from the PMA results are presented in Figure 3 and Table S3 (File S5). All therapies except THP are more effective than TURBT (no further treatment) in preventing recurrence. BCG, MMC, and IFN are more effective than TURBT with reference to progression. Compared with BCG, therapies such as ADM, EPI, IFN, and THP are less effective in preventing recurrence. With regard to progression, however, there are no significant differences in efficacy between intravesical therapies, except in the comparison between MMC and ADM. Substantial heterogeneity is observed in seven recurrence comparisons (ADM‐TURBT, BCG‐ADM, BCG‐EPI, BCG‐GEM, BCG‐MMC, MMC‐IFN, and THP‐TURBT). All the progression comparisons reveal low or moderate heterogeneities. Subgroup and sensitivity analysis is performed based on potential factors (Table S4). Generally, the effect sizes in subgroup analyses are similar to those in direct comparisons.

### 3.3 Network meta‐analysis results

**FIGURE 4:**
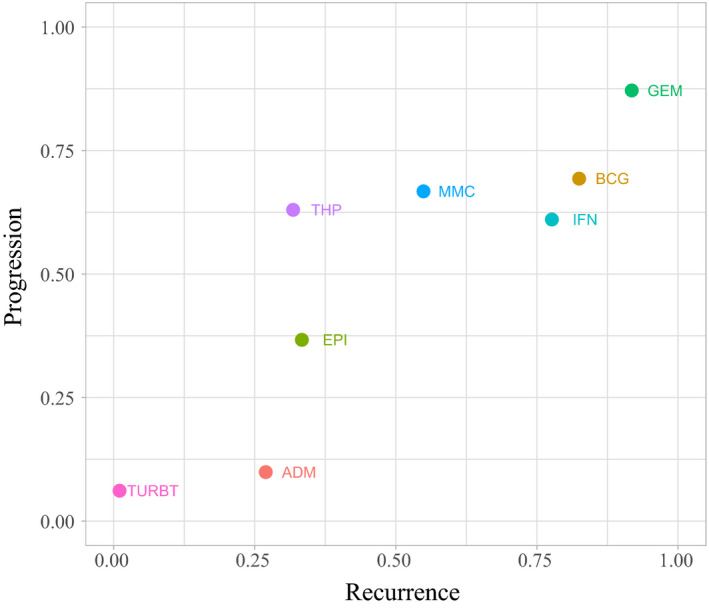
SUCRA plot for ranking for recurrence and progression prevention

Efficacy estimates calculated from NMA are listed in Table 1. ADM fails to prevent progression when compared with TURBT (HR: 0.94, 95%CrI: 0.66–1.35). Regarding recurrence, BCG is more effective than all the other therapies excluding GEM (BCG vs. GEM: HR: 1.21, 95%CrI: 0.73–1.97). There are no significant differences between GEM, IFN, MMC, and THP, and BCG, with regard to progression. Among the 19 recurrence comparisons, only 3 comparisons show inconsistency (EPI‐BCG, MMC‐BCG, TURBT‐EPI) (Table S5). Only one comparison (TURBT‐MMC) exhibits inconsistency out of 17 comparisons for progression. Year, sample size, center, adverse effect, quality, and grade subgroups yield similar results with overall NMA (Tables S6 and S7). Network meta‐regression reveals no significant relationship between outcomes and study characteristics (Table S8). The ranking probabilities for 8 interventions are listed in Tables S9 and S10. GEM is the most effective therapy considering both recurrence and progression outcomes (Figure 4, Figures S3 and S4), whereas the efficacy of BCG and IFN is comparable. However, ADM efficacy is poor in tumor progression prevention.

**TABLE 1:** Efficacy estimates table from network meta‐analysis with 95% credible intervals

**ADM**	2.69 [1.80 to 3.97]	1.86 [1.21 to 2.83]	3.42 [1.84 to 6.69]	2.46 [1.23 to 5.00]	2.61 [1.80 to 3.86]	2.59 [0.77 to 9.30]	0.94 [0.66 to 1.35]
1.65 [1.28 to 2.12]	**BCG**	0.69 [0.50 to 0.93]	1.28 [0.76 to 2.16]	0.90 [0.49 to 1.73]	0.98 [0.76 to 1.26]	0.96 [0.29 to 3.53]	0.35 [0.26 to 0.48]
1.05 [0.78 to 1.43]	0.64 [0.48 to 0.84]	**EPI**	1.86 [1.02 to 3.35]	1.31 [0.67 to 2.72]	1.42 [0.99 to 2.08]	1.39 [0.41 to 5.26]	0.51 [0.35 to 0.74]
1.97 [1.14 to 3.42]	1.21 [0.73 to 1.97]	1.88 [1.06 to 3.32]	**GEM**	0.71 [0.26 to 1.63]	0.76 [0.47 to 1.28]	0.74 [0.20 to 2.97]	0.27 [0.15 to 0.50]
1.58 [1.09 to 2.32]	0.96 [0.67 to 1.39]	1.51 [0.99 to 2.29]	0.79 [0.44 to 1.48]	**IFN**	1.08 [0.55 to 2.03]	1.05 [0.26 to 4.39]	0.39 [0.20 to 0.70]
1.27 [1.01 to 1.62]	0.78 [0.63 to 0.95]	1.21 [0.90 to 1.65]	0.64 [0.38 to 1.08]	0.81 [0.57 to 1.14]	**MMC**	0.98 [0.29 to 3.60]	0.36 [0.25 to 0.51]
1.01 [0.61 to 1.70]	0.62 [0.36 to 0.95]	0.96 [0.55 to 1.70]	0.51 [0.25 to 1.05]	0.64 [0.35 to 1.15]	0.79 [0.47 to 1.35]	**THP**	0.36 [0.10 to 1.26]
0.70 [0.57 to 0.85]	0.43 [0.34 to 0.54]	0.66 [0.50 to 0.89]	0.35 [0.21 to 0.61]	0.44 [0.31 to 0.63]	0.55 [0.44 to 0.68]	0.69 [0.41 to 1.14]	**TURBT**

Note

Effect sizes below the diagonal refer to recurrence outcome and effect sizes above the diagonal refer to progression outcome. Effect sizes greater than 0 favor the therapy on the left or above and effect sizes less than 0 favor the therapy on the right or below.

### 3.4 Evidence summary

Of 28 comparisons, nine recurrence outcome comparisons are based on indirect evidence, and 11 progression outcome comparisons are based on indirect evidence (Table 2). The confidence in estimated effect sizes ranges from very low to moderate. Study limitations are major reasons for the downgrading of the confidence of evidence. The reliability of the therapeutic rank with regard to recurrence is low due to study limitations and inconsistency. Similarly, with regard to progression, the reliability of ranking is very low due to study limitations, indirectness, and imprecision.

**TABLE 2:** Summary of confidence in estimated effect sizes and therapy ranking

Comparison	Recurrence			Progression		
	Evidence type	Confidence	Downgrading reasons	Evidence type	Confidence	Downgrading reasons
BCG versus MMC	Mixed	Very low	Sl; Id; Ic	Mixed	Low	Sl; Ip
BCG versus IFN	Mixed	Low	Sl; Ip	Mixed	Low	Sl; Ip
BCG versus ADM	Mixed	Low	Sl; Ic	Mixed	Moderate	Sl
BCG versus EPI	Mixed	Low	Sl; Ic	Mixed	Moderate	Sl;
BCG versus GEM	Mixed	Low	Sl; Ic	Mixed	Low	Sl; Ip
BCG versus THP	Mixed	Moderate	Sl;	Mixed	Low	Sl; Ip
BCG versus TURBT	Mixed	Moderate	Sl;	Mixed	Moderate	Sl;
MMC versus IFN	Mixed	Very low	Sl; Ic; Ip	Mixed	Low	Sl; Ip
MMC versus ADM	Mixed	Low	Sl; Id	Mixed	Moderate	Sl;
MMC versus EPI	Mixed	Low	Sl; Ip	Mixed	Low	Sl; Ip
MMC versus GEM	Mixed	Low	Sl; Ip	Mixed	Low	Sl; Ip
MMC versus THP	Mixed	Low	Sl; Ip	Mixed	Low	Sl; Ip
MMC versus TURBT	Mixed	Moderate	Sl	Mixed	Low	Sl; Ic
IFN versus ADM	Indirect	Low	Sl; Id	Indirect	Low	Sl; Id
IFN versus EPI	Indirect	Very low	Sl; Id; Ip	Indirect	Very low	Sl; Id; Ip
IFN versus GEM	Indirect	Very low	Sl; Id; Ip	Indirect	Very low	Sl; Id; Ip
IFN versus THP	Indirect	Very low	Sl; Id; Ip	Indirect	Very low	Sl; Id; Ip
IFN versus TURBT	Mixed	Moderate	Sl;	Mixed	Moderate	Sl;
ADM versus EPI	Mixed	Low	Sl; Ip	Mixed	Moderate	Sl;
ADM versus GEM	Indirect	Low	Sl; Id;	Indirect	Low	Sl; Id;
ADM versus THP	Mixed	Low	Sl; Ip	Mixed	Low	Sl; Ip
ADM versus TURBT	Mixed	Very low	Sl; Ic; Pb	Mixed	Low	Sl; Ip
EPI versus GEM	Indirect	Low	Sl; Id;	Indirect	Moderate	Sl
EPI versus THP	Indirect	Very low	Sl; Id; Ip	Indirect	Low	Sl; Ip
EPI versus TURBT	Mixed	Low	Sl; Ic;	Indirect	Low	Sl; Id
GEM versus THP	Indirect	Very low	Sl; Id; Ip	Indirect	Very low	Sl; Id; Ip
GEM versus TURBT	Indirect	Low	Sl; Id;	Indirect	Low	Sl; Id
THP versus TURBT	Mixed	Very low	Sl; Ic; Ip	Indirect	Low	Sl; Ip
Therapy ranking		Low	Sl; Ic		Very low	Sl; Id; Ip

Abbreviations: Ic, inconsistency; Id, indirectness; Ip, imprecision; Pb, publication bias; Sl, study limitations.

## 4 DISCUSSION

The present NMA comprehensively compares the preventive effects of common intravesical instillation monotherapies on the recurrence and progression of non‐muscle‐invasive bladder cancer. GEM has the greatest potential to be the optimal therapy, and IFN could be the alternative therapy to BCG due to their similar efficacy. However, ADM efficacy on the prevention of progression is limited. Our findings provide a potential solution to the global BCG shortage, and could help urologists select appropriate intravesical therapies for NMIBC patients in the absence of BCG, or when it is contraindicated or there is BCG intolerance.

Based on current direct and indirect evidence, GEM is ranked higher than BCG in efficacy. Three RCTs compared GEM and BCG are included.^24^, ^25^, ^26^ GEM was reported to be inferior to Tice BCG strain in patients with high‐risk NMIBC.^24^ Nevertheless, GEM seems to have higher recurrence‐free rates than Connaught BCG strain in NMIBC patients with high risk of recurrence and BCG failure NMIBC.^25^ In another study, there was no difference in recurrence and progression between GEM and 1/3 Connaught BCG strain dose in patients with intermediate risk NMIBC.^26^ A case‐controlled study revealed that GEM was associated with disease‐free survival superior to that of BCG, independent of tumor grade, risk, prior BCG, and number of tumors.^27^ Although the studies available for comparing BCG and GEM are limited, current evidence highlights the promising efficacy of GEM in preventing NMIBC recurrence. Large‐scale trials should be conducted on the efficacy of GEM as an alternative drug to BCG.

IFN, in addition to BCG, has been demonstrated to have potent prophylaxis effects on NMIBC recurrence and progression. Our study includes both IFN‐α and IFN‐γ, and we observe that IFN is slightly inferior to BCG in ranking. Only one RCT compared the effectiveness of BCG and recombinant IFN‐α‐2b directly in recurrent T1 tumor.^28^ The recurrence rate in a 2‐year follow‐up in the IFN group (69.4%) was higher than that in the BCG group (39.3%).^28^ Subgroup analysis of differences in tumor grade also indicates increasing recurrence rates in the IFN group: grade 1 (64.5% vs. 29%), grade 2 (66.6% vs. 46%), and grade 3 (100% vs. 75%). However, another study reported a recurrence rate of only 22%–36% in patients with Ta/1G2 tumor following IFN‐α‐2b therapy, when compared with TURBT.^29^ The NMA results complement the limited evidence by combining direct and indirect evidence. Mixed results exhibited comparable efficacy between BCG and IFN (Table 1). Recent studies have also confirmed the efficacy of IFN‐based gene therapy, recombinant adenovirus‐mediated interferon‐α‐2b (rAd‐IFN‐α‐2b).^30^ It can enhance the effect of IFN by prolonging its duration. However, more evidence is required to demonstrate the robustness of the efficacy of the IFN‐based therapy.

BCG and MMC are similar, with no significant difference between the two therapies in PMA with regard to both recurrence and progression. A recent Cochrane review compared the efficacy of BCG and MMC in Ta and T1 bladder cancer based on HR.^31^ Although the pooled effect sizes based on time to recurrence, progression, and death favor BCG, their differences were non‐significant.^31^ However, the NMA results in this study indicate that BCG is significantly more effective than MMC, which shows inconsistency of the comparison. In two other NMAs, conversely, the authors did not observe any differences between BCG and MMC based on odds ratio effect sizes.^13^, ^14^ In subgroup analysis for PMA in the present study, we observe that MMC and BCG schedules could influence their efficacy. Maintenance BCG has recurrence outcomes superior to maintenance MMC outcomes, whereas the efficacy of induction BCG is comparable to that of maintenance or induction MMC. The NMA results were similar: network subgroup analysis also demonstrates that maintenance BCG is more effective than maintenance MMC. An early meta‐analysis compared maintenance/induction BCG with MMC using HR.^32^ The authors reported that maintenance BCG was superior to maintenance MMC; however, non‐maintenance BCG had significantly poor efficacy of prophylaxis based on recurrence than MMC.^32^ Notably, we did not include chemohyperthermia of MMC because it is still under investigation.^33^, ^34^

BCG instillation is associated with high incidence rates of adverse events. The rate of withdrawal is 9.9% to 52.5% due to toxicity in the course of maintenance BCG instillation.^35^ Nevertheless, adverse events that caused by chemotherapy are rare. MMC generated fewer local toxicities (30% vs. 44%) and systemic toxicities than BCG (12% vs. 19%).^31^ IFN instillation was well tolerated, and no complications were reported in numerous studies.^28^, ^29^, ^36^, ^37^ GEM seems to be more tolerated than BCG. A phase Ⅱ trial compared GEM with 1/3 dose BCG and indicated that more frequent local and systemic side effects were observed in BCG group (56.1% vs. 35.7%).^26^ Another RCT also found that no patient in GEM group needed postponed treatment in but six in 32 patients suspended after receiving BCG treatment.^24^ Specifically, BCG therapy was more likely to cause bladder irrigation sign, such as dysuria and hematuria.^25^, ^26^, ^38^ Rare symptoms other than urinary system, such as dermatitis and nausea/vomiting were reported during GEM treatment.^25^ Therefore, GEM, IFN, and MMC could be appropriate second‐line treatments with favorable efficacy and excellent safety when in the case of BCG intolerance.

The present study has several limitations. The major limitation is that the majority of included RCTs have moderate to high risk of bias, which decreases the reliability of the evidence used in treatment ranking. The reasons for the low RCT qualities are unspecified randomization processes, unblind study designs, and missing outcome data. More well‐designed randomized controlled trials are required to enhance the reliability of the findings of NMAs. Second, the heterogeneity between studies regarding recurrence is generally high. Network meta‐regression was performed but fails to reveal any impact of potential factors on efficacy. Besides, the differences between most progression comparisons are not significant, which leads to potentially an imprecision in ranking probability. The bias could arise from the small and comparable number of patients who developed tumor progression between treatment arms. Finally, the dose of therapy varies from cohorts, which may affect the pooled results. It is difficult to conduct a subgroup and sensitivity analysis for drug dose. However, results from previous studies show that different doses of drugs yielded similar effects in preventing tumor recurrence or progression.^14^, ^39^, ^40^ Therefore, we assume that the dose had a limited impact on the therapeutic rank.

## 5 CONCLUSIONS

This study comprehensively evaluates the efficacy of intravesical instillation monotherapy in preventing NMIBC recurrence and progression. The therapeutic rank of GEM was superior to that of BCG in the network model. IFN and MMC are the optional second‐line therapies. However, the results of this study should be treated with caution since most of the included studies are of moderate to high risk of bias.

## CONFLICT OF INTEREST

The authors have no conflict of interest to disclose.

## Supporting information

File S1

File S2

File S3

File S4

File S5

## Data Availability

The data used in the work can be obtained in supplementary file 4.
