# Corrigendum

**DOI:** 10.1002/cam4.4174

**Published:** 2021-08-16

In the article by Lu J‐L et al,^1^ the authors recently found two errors in the published version.

In Table 1, the footnote should read “Effect sizes below the diagonal refer to recurrence outcome and effect sizes above the diagonal refer to progression outcome. Effect sizes greater than 1 favor the therapy on the left or above and effect sizes less than 1 favor the therapy on the right or below.”

In Table S10, the data of rank 1 to rank 8 is similar to that in Table S9 (recurrence). The error has been corrected in the revised Table S10.

The authors apologize for the unintentional errors that may have misled readers. The errors had no impact on the interpretation and conclusion of this work.
