# Supplementary material for: Efficacy of intravesical therapies on the prevention of recurrence and progression of non‐muscle‐invasive bladder cancer: A systematic review and network meta‐analysis
Source: Cancer Med. 2020 Oct 11;9(21):7800–9. doi: 10.1002/cam4.3513 (PMC7643689; doi:10.1002/cam4.3513)
Supplement: Supplementary file 2 — File S2 [file CAM4-9-7800-s002.docx]

**Search strategy in Ovid Medline**

| Step | Search term |
| --- | --- |
| 1 | exp bladder cancer/ |
| 2 | ((((non or "not") adj (invas* or invad* or infiltrat*)) or noninvas* or noninvad* or noninfiltrat*) adj5 muscle*).ti,ab. |
| 3 | (cis or Tis or ta or t1* or superficial).ti,ab. |
| 4 | 2 or 3 |
| 5 | 1 and 4 |
| 6 | exp mitomycin/ |
| 7 | mitomycin.ti,ab. |
| 8 | exp Mycobacterium bovis BCG/ |
| 9 | (Bacillus Calmette Guerin or BCG).ti,ab. |
| 10 | exp gemcitabine/ |
| 11 | (gemcitabine or GEM).ti,ab. |
| 12 | exp thiotepa/ |
| 13 | thiotepa.ti,ab. |
| 14 | exp doxorubicin/ |
| 15 | (doxorubicin OR adriamycin OR DOX OR ADM).ti,ab. |
| 16 | exp interferon/ |
| 17 | (interferon* or INF* or Ad-IFN).ti,ab. |
| 18 | exp epirubicin/ |
| 19 | (epirubicin or EPI).ti,ab. |
| 20 | 6 or 7 or 8 or 9 or 10 or 11 or 12 or 13 or 14 or 15 or 16 or 17 or 18 or 19 |
| 21 | 5 and 20 |
