# Supplementary material for: Efficacy of intravesical therapies on the prevention of recurrence and progression of non‐muscle‐invasive bladder cancer: A systematic review and network meta‐analysis
Source: Cancer Med. 2020 Oct 11;9(21):7800–9. doi: 10.1002/cam4.3513 (PMC7643689; doi:10.1002/cam4.3513)
Supplement: Supplementary file 3 — File S3 [file CAM4-9-7800-s003.docx]

**Excluded studies with reasons**

One hundred and four studies were excluded after full-text screening. The table below lists the title, first author, publication year and the exclusion reasons of the studies. (Abbreviation: II, immediate instillation; CT, combined therapy; ND, no follow-up data; SD, single drug study; ROT, reports on one trial)

| Author | Year | Title | Reason |
| --- | --- | --- | --- |
| Burnand | 1976 | Single Dose Intravesical Thiotepa as an Adjuvant to Cystodiathermy in the Treatment of Transitional Cell Bladder Carcinoma | II |
| Abrams | 1981 | A Controlled Trial of Single Dose lntravesical Adriamycin in Superficial Bladder Tumours | II |
| Koontz | 1981 | The Use of Intravesical Thio-Tepa in the Management of Non-Invasive Carcinoma of the Bladder | II |
| Zincke | 1985 | Intravesical Thiotepa and Mitomycin C Treatment Immediately After Transurethral Resection and Later for Superficial (Stages Ta and Tis) Bladder Cancer: A Prospective, Randomized, Stratified Study With Crossover Design | II |
| Oosterlinck | 1993 | A Prospective European Organization for Research and Treatment of Cancer Genitourinary Group Randomized Trial Comparing Transurethral Resection Followed by a Single Intravesical Instillation of Epirubicin or Water in Single Stage Ta, T1 Papillary Carcinoma of the Bladder | II |
| Solsona | 1999 | Effectiveness of a Single Immediate Mitomycin C Instillation in Patients With Low Risk Superficial Bladder Cancer: Short and Long-Term Followup | II |
| Rajala | 1999 | Transurethral Resection With Perioperative Instilation on Interferon-Alpha or Epirubicin for the Prophylaxis of Recurrent Primary Superficial Bladder Cancer: A Prospective Randomized Multicenter study--Finnbladder III | II |
| Rajala | 2002 | Perioperative Single Dose Instillation of Epirubicin or Interferon-Alpha After Transurethral Resection for the Prophylaxis of Primary Superficial Bladder Cancer Recurrence: A Prospective Randomized Multicenter study--FinnBladder III Long-Term Results | II |
| Okamura | 2002 | Randomized Study of Single Early Instillation of (2"R)-4'-O-tetrahydropyranyl-doxorubicin for a Single Superficial Bladder Carcinoma | II |
| Barghi | 2006 | Immediate Intravesical Instillation of Mitomycin C After Transurethral Resection of Bladder Tumor in Patients With Low-Risk Superficial Transitional Cell Carcinoma of Bladder | II |
| El-Ghobashy | 2007 | Effectiveness of a Single Immediate Mitomycin C Instillation in Patients With Low Risk Superficial Bladder Cancer: Short and Long-Term Follow-Up | II |
| Berrum-Svennung | 2008 | A Single Instillation of Epirubicin After Transurethral Resection of Bladder Tumors Prevents Only Small Recurrences | II |
| Cai | 2008 | Can Early Single Dose Instillation of Epirubicin Improve Bacillus Calmette-Guerin Efficacy in Patients With Nonmuscle Invasive High Risk Bladder Cancer? Results From a Prospective, Randomized, Double-Blind Controlled Study | II |
| Gudjónsson | 2009 | Should All Patients With Non-Muscle-Invasive Bladder Cancer Receive Early Intravesical Chemotherapy After Transurethral Resection? The Results of a Prospective Randomised Multicentre Study | II |
| Böhle | 2009 | Single Postoperative Instillation of Gemcitabine in Patients With Non-Muscle-Invasive Transitional Cell Carcinoma of the Bladder: A Randomised, Double-Blind, Placebo-Controlled Phase III Multicentre Study | II |
| Türkeri | 2010 | Comparison of the Efficacy of Single or Double Intravesical Epirubicin Instillation in the Early Postoperative Period to Prevent Recurrences in Non-Muscle-Invasive Urothelial Carcinoma of the Bladder: Prospective, Randomized Multicenter Study | II |
| Saika | 2010 | Two Instillations of Epirubicin as Prophylaxis for Recurrence After Transurethral Resection of Ta and T1 Transitional Cell Bladder Cancer: A Prospective, Randomized Controlled Study | II |
| Onishi | 2011 | Continuous Saline Bladder Irrigation After Transurethral Resection Is a Prophylactic Treatment Choice for Non-Muscle Invasive Bladder Tumor | II |
| Di Stasi | 2011 | Electromotive Instillation of Mitomycin Immediately Before Transurethral Resection for Patients With Primary Urothelial Non-Muscle Invasive Bladder Cancer: A Randomised Controlled Trial | II |
| De Nunzio | 2011 | Long-term Experience With Early Single Mitomycin C Instillations in Patients With Low-Risk Non-Muscle-Invasive Bladder Cancer: Prospective, Single-Centre Randomised Trial | II |
| Li | 2013 | Efficacy of Immediate Instillation Combined With Regular Instillations of Pirarubicin for Ta and T1 Transitional Cell Bladder Cancer After Transurethral Resection: A Prospective, Randomized, Multicenter Study | II |
| Ersoy | 2013 | Single Early Instillation of Mitomycin C and Urinary Alkalinization in Low-Risk Non-Muscle-Invasive Bladder Cancer: A Preliminary Study | II |
| Karsh | 2018 | Double-Blind, Randomized, Placebo-controlled Studies Evaluating Apaziquone (E09, Qapzola™) Intravesical Instillation Post Transurethral Resection of Bladder Tumors for the Treatment of Low-risk Non-Muscle Invasive Bladder Cancer | II |
| Messing | 2018 | Effect of Intravesical Instillation of Gemcitabine vs Saline Immediately Following Resection of Suspected Low-Grade Non-Muscle-Invasive Bladder Cancer on Tumor Recurrence: SWOG S0337 Randomized Clinical Trial | II |
| Tanimoto | 2018 | Prospective Randomized Controlled Trial of Postoperative Early Intravesical Chemotherapy With Pirarubicin (THP) for Solitary Non-Muscle Invasive Bladder Cancer Comparing Single and Two-Time Instillation | II |
| Naya | 2018 | Randomized Study of Intravesical Pirarubicin Chemotherapy With Low and Intermediate-Risk Nonmuscle-Invasive Bladder Cancer in Japan: Comparison of a Single Immediate Postoperative Intravesical Instillation With Short-Term Adjuvant Intravesical Instillations After Transurethral Resection | II |
| Elsawy | 2019 | The Value of Immediate Postoperative Intravesical Epirubicin Instillation as an Adjunct to Standard Adjuvant Treatment in Intermediate and High-Risk Non-Muscle-Invasive Bladder Cancer: A Preliminary Results of Randomized Controlled Trial | II |
| Shore | 2017 | Intravesical rAd-IFNα/Syn3 for Patients With High-Grade, Bacillus Calmette-Guerin-Refractory or Relapsed Non-Muscle-Invasive Bladder Cancer: A Phase II Randomized Study | II |
| Onishi | 2017 | Randomized Controlled Study of the Efficacy and Safety of Continuous Saline Bladder Irrigation After Transurethral Resection for the Treatment of Non-Muscle-Invasive Bladder Cancer | II |
| Schulman | 1978 | Adjuvant Therapy of T1 Bladder Carcinoma: Preliminary Results of an EORTC Randomized Study | ND |
| Fukui | 1992 | Intravesical Combination Chemotherapy With Mitomycin C and Doxorubicin for Superficial Bladder Cancer: A Randomized Trial of Maintenance Versus No Maintenance Following a Complete Response | CT |
| Rintala | 1996 | Alternating Mitomycin C and Bacillus Calmette-Guerin Instillation Prophylaxis for Recurrent Papillary (Stages Ta to T1) Superficial Bladder Cancer. Finnbladder Group | CT |
| Witjes | 1998 | Results of a Randomized Phase III Trial of Sequential Intravesical Therapy With Mitomycin C and Bacillus Calmette-Guerin Versus Mitomycin C Alone in Patients With Superficial Bladder Cancer | CT |
| Ali-El-Dein | 1999 | Sequential Bacillus Calmette-Guerin and Epirubicin Versus Bacillus Calmette-Guerin Alone for Superficial Bladder Tumors: A Randomized Prospective Study | CT |
| Bilen | 2000 | Clinical Experience With BCG Alone Versus BCG Plus Epirubicin | CT |
| Kaasinen | 2000 | Weekly Mitomycin C Followed by Monthly Bacillus Calmette-Guerin or Alternating Monthly interferon-alpha2B and Bacillus Calmette-Guerin for Prophylaxis of Recurrent Papillary Superficial Bladder Carcinoma | CT |
| Sekine | 2001 | Equivalent Efficacy of Mitomycin C Plus Doxorubicin Instillation to Bacillus Calmette-Guerin Therapy for Carcinoma in Situ of the Bladder | CT |
| Kaasinen | 2003 | Alternating Mitomycin C and BCG Instillations Versus BCG Alone in Treatment of Carcinoma in Situ of the Urinary Bladder: A Nordic StudyT | CT |
| Di Stasi | 2003 | Intravesical Electromotive Mitomycin C Versus Passive Transport Mitomycin C for High Risk Superficial Bladder Cancer: A Prospective Randomized Study | CT |
| Di Stasi | 2006 | Sequential BCG and Electromotive Mitomycin Versus BCG Alone for High-Risk Superficial Bladder Cancer: A Randomised Controlled Trial | CT |
| Cho | 2009 | The Effects of Intravesical Chemoimmunotherapy With Gemcitabine and Bacillus Calmette-Guérin in Superficial Bladder Cancer: A Preliminary Study | CT |
| Nepple | 2010 | Bacillus Calmette-Guérin With or Without Interferon α-2b and Megadose Versus Recommended Daily Allowance Vitamins During Induction and Maintenance Intravesical Treatment of Nonmuscle Invasive Bladder Cancer | CT |
| Duchek | 2010 | Bacillus Calmette-Guérin Is Superior to a Combination of Epirubicin and interferon-alpha2b in the Intravesical Treatment of Patients With Stage T1 Urinary Bladder Cancer. A Prospective, Randomized, Nordic Study | CT |
| El Mohsen | 2010 | Sequential Chemoimmunotherapy Using Mitomycin Followed by Bacillus Calmette-Guerin (MCC + BCG) Versus Single-Agent Immunotherapy (BCG) for Recurrent Superfcial Bladder Tumors | CT |
| Chiong | 2011 | NRAMP1 and hGPX1 Gene Polymorphism and Response to Bacillus Calmette-Guérin Therapy for Bladder Cancer | CT |
| Oosterlinck | 2011 | Sequential Intravesical Chemoimmunotherapy With Mitomycin C and Bacillus Calmette-Guérin and With Bacillus Calmette-Guérin Alone in Patients With Carcinoma in Situ of the Urinary Bladder: Results of an EORTC Genito-Urinary Group Randomized Phase 2 Trial (30993) | CT |
| Järvinen | 2012 | Long-term Results of Maintenance Treatment of Mitomycin C or Alternating Mitomycin C and Bacillus Calmette-Guérin Instillation Therapy of Patients With Carcinoma in Situ of the Bladder: A Subgroup Analysis of the Prospective FinnBladder 2 Study With a 17-year Follow-Up | CT |
| Gülpinar | 2012 | The Value of Perioperative Mitomycin C Instillation in Improving Subsequent Bacillus Calmette-Guerin Instillation Efficacy in Intermediate and High-Risk Patients With Non-Muscle Invasive Bladder Cancer: A Prospective Randomized Study | CT |
| Hemdan | 2014 | 5-Year Outcome of a Randomized Prospective Study Comparing Bacillus Calmette-Guérin With Epirubicin and interferon-α2b in Patients With T1 Bladder Cancer | CT |
| Solsona | 2015 | Sequential Combination of Mitomycin C Plus Bacillus Calmette-Guérin (BCG) Is More Effective but More Toxic Than BCG Alone in Patients With Non-Muscle-Invasive Bladder Cancer in Intermediate- And High-Risk Patients: Final Outcome of CUETO 93009, a Randomized Prospective Trial | CT |
| Marttila | 2016 | Intravesical Bacillus Calmette-Guérin Versus Combination of Epirubicin and Interferon-α2a in Reducing Recurrence of Non-Muscle-invasive Bladder Carcinoma: FinnBladder-6 Study | CT |
| Glashan | 1990 | A Randomized Controlled Study of Intravesical alpha-2b-interferon in Carcinoma in Situ of the Bladder | SD |
| Hoeltl | 1991 | How Effective Is Topical alpha-2b Interferon in Preventing Recurrence of Superficial Bladder Cancer? | SD |
| Ueda | 1992 | Adjuvant Chemotherapy With Early Intravesical Instillation of Adriamycin and Long-Term Oral Administration of 5-fluorouracil in Superficial Bladder Cancer. The Kyushu University Urological Oncology Groupt | SD |
| Morales | 1992 | Dose-response of Bacillus Calmette-Guerin in the Treatment of Superficial Bladder Cancer | SD |
| Akaza | 1995 | Bacillus Calmette-Guérin Treatment of Existing Papillary Bladder Cancer and Carcinoma in Situ of the Bladder. Four-year Results. The Bladder Cancer BCG Study Group | SD |
| Bouffioux | 1995 | Intravesical Adjuvant Chemotherapy for Superficial Transitional Cell Bladder Carcinoma: Results of 2 European Organization for Research and Treatment of Cancer Randomized Trials With Mitomycin C and Doxorubicin Comparing Early Versus Delayed Instillations and Short-Term Versus Long-Term Treatment. European Organization for Research and Treatment of Cancer Genitourinary Group | SD |
| Gruenwald | 1997 | A 12 Versus 6-week Course of Bacillus Calmette-Guerin Prophylaxis for the Treatment of High Risk Superficial Bladder Cancer | SD |
| Okamura | 1998 | A Randomized Study of Short-Versus Long-Term Intravesical Epirubicin Instillation for Superficial Bladder Cancer. Nagoya University Urological Oncology Group | SD |
| Okamura | 1998 | A Randomized Study of Short-Versus Long-Term Intravesical Epirubicin Instillation for Superficial Bladder Cancer. Nagoya University Urological Oncology Group | SD |
| Lamm | 2000 | Maintenance Bacillus Calmette-Guerin Immunotherapy for Recurrent TA, T1 and Carcinoma in Situ Transitional Cell Carcinoma of the Bladder: A Randomized Southwest Oncology Group Study | SD |
| Palou | 2001 | Control Group and Maintenance Treatment With Bacillus Calmette-Guerin for Carcinoma in Situ and/or High Grade Bladder Tumors | SD |
| Au | 2001 | Methods to Improve Efficacy of Intravesical Mitomycin C: Results of a Randomized Phase III Trial | SD |
| Nomata | 2002 | Intravesical Adjuvant Chemotherapy for Superficial Transitional Cell Bladder Carcinoma: Results of a Randomized Trial With Epirubicin Comparing Short-Term Versus Long-Term Maintenance Treatment | SD |
| Martínez-Piñeiro | 2002 | Long-term Follow-Up of a Randomized Prospective Trial Comparing a Standard 81 Mg Dose of Intravesical Bacille Calmette-Guérin With a Reduced Dose of 27 Mg in Superficial Bladder Cancer | SD |
| Irie | 2003 | Sufficient Prophylactic Efficacy With Minor Adverse Effects by Intravesical Instillation of Low-Dose Bacillus Calmette-Guérin for Superficial Bladder Cancer Recurrence | SD |
| Koga | 2004 | A Randomized Controlled Trial of Short-Term Versus Long-Term Prophylactic Intravesical Instillation Chemotherapy for Recurrence After Transurethral Resection of Ta/T1 Transitional Cell Carcinoma of the Bladder | SD |
| Mitsumori | 2004 | Early and Large-Dose Intravesical Instillation of Epirubicin to Prevent Superficial Bladder Carcinoma Recurrence After Transurethral Resection | SD |
| Kuroda | 2004 | Effect of Prophylactic Treatment With Intravesical Epirubicin on Recurrence of Superficial Bladder cancer--The 6th Trial of the Japanese Urological Cancer Research Group (JUCRG): A Randomized Trial of Intravesical Epirubicin at Dose of 20mg/40ml, 30mg/40ml, 40mg/40ml | SD |
| Martínez-Piñeiro | 2005 | Has a 3-fold Decreased Dose of Bacillus Calmette-Guerin the Same Efficacy Against Recurrences and Progression of T1G3 and Tis Bladder Tumors Than the Standard Dose? Results of a Prospective Randomized Trial | SD |
| Gårdmark | 2005 | Randomized Phase II Marker Lesion Study Evaluating Effect of Scheduling on Response to Intravesical Gemcitabine in Recurrent Stage Ta Urothelial Cell Carcinoma of the Bladder | SD |
| Lerner | 2007 | Patterns of Recurrence and Outcomes Following Induction Bacillus Calmette-Guerin for High Risk Ta, T1 Bladder Cancer | SD |
| Hendricksen | 2007 | Comparison of Three Schedules of Intravesical Epirubicin in  Patients with Non–Muscle-Invasive Bladder Cancer | SD |
| Serretta | 2010 | doi: 10.1111/j.1464-410X.2009.09153.x. Epub 2010 Jan 11.  A 1-year Maintenance After Early Adjuvant Intravesical Chemotherapy Has a Limited Efficacy in Preventing Recurrence of Intermediate Risk Non-Muscle-Invasive Bladder Cancer | SD |
| Koga | 2010 | Maintenance Intravesical Bacillus Calmette-Guérin Instillation for Ta, T1 Cancer and Carcinoma in Situ of the Bladder: Randomized Controlled Trial by the BCG Tokyo Strain Study Group | SD |
| Colombo | 2012 | Neoadjuvant Short-Term Intensive Intravesical Mitomycin C Regimen Compared With Weekly Schedule for Low-Grade Recurrent Non-Muscle-Invasive Bladder Cancer: Preliminary Results of a Randomised Phase 2 Study | SD |
| Sengiku | 2013 | A Prospective Comparative Study of Intravesical Bacillus Calmette-Guérin Therapy With the Tokyo or Connaught Strain for Nonmuscle Invasive Bladder Cancer | SD |
| Oddens | 2013 | Final Results of an EORTC-GU Cancers Group Randomized Study of Maintenance Bacillus Calmette-Guérin in Intermediate- And High-Risk Ta, T1 Papillary Carcinoma of the Urinary Bladder: One-Third Dose Versus Full Dose and 1 Year Versus 3 Years of Maintenance | SD |
| Pfister | 2015 | Efficacy and Tolerance of One-Third Full Dose Bacillus Calmette-Guérin Maintenance Therapy Every 3 Months or 6 Months: Two-Year Results of URO-BCG-4 Multicenter Study | SD |
| Brausi | 2014 | Side Effects of Bacillus Calmette-Guérin (BCG) in the Treatment of Intermediate- And High-Risk Ta, T1 Papillary Carcinoma of the Bladder: Results of the EORTC Genito-Urinary Cancers Group Randomised Phase 3 Study Comparing One-Third Dose With Full Dose and 1 Year With 3 Years of Maintenance BCG | SD |
| Martínez-Piñeiro | 2015 | Maintenance Therapy With 3-monthly Bacillus Calmette-Guérin for 3 Years Is Not Superior to Standard Induction Therapy in High-risk Non-muscle-invasive Urothelial Bladder Carcinoma: Final Results of Randomised CUETO Study 98013 | SD |
| Nakai | 2016 | Insignificant Role of Bacillus Calmette-Guérin Maintenance Therapy After Complete Transurethral Resection of Bladder Tumor for Intermediate- And High-Risk Non-Muscle-Invasive Bladder Cancer: Results From a Randomized Trial | SD |
| Nouhaud | 2017 | Final Results of the Phase III URO-BCG 4 Multicenter Study: Efficacy and Tolerance of One-Third Dose BCG Maintenance in Nonmuscle Invasive Bladder Cancer | SD |
| Yokomizo | 2016 | Randomized Controlled Study of the Efficacy, Safety and Quality of Life With Low Dose Bacillus Calmette-Guérin Instillation Therapy for Nonmuscle Invasive Bladder Cancer | SD |
| Badalament | 1987 | A Prospective Randomized Trial of Maintenance Versus Nonmaintenance Intravesical Bacillus Calmette-Guérin Therapy of Superficial Bladder Cancer | SD |
| Hudson | 1987 | Single Course Versus Maintenance Bacillus Calmette-Guerin Therapy for Superficial Bladder Tumors: A Prospective, Randomized Trial | SD |
| Flamm | 1990 | Long-term Versus Short-Term Doxorubicin Hydrochloride Instillation After Transurethral Resection of Superficial Bladder Cancer | SD |
| Niijima | 1983 | Randomized Clinical Trial on Chemoprophylaxis of Recurrence in Cases of Superficial Bladder Cancer | ROT |
| MRC | 1985 | The Effect of Intravesical Thiotepa on the Recurrence Rate of Newly Diagnosed Superficial Bladder Cancer. An MRC Study. MRC Working Party on Urological Cancer | ROT |
| Herr | 1988 | Bacillus Calmette-Guérin Therapy Alters the Progression of Superficial Bladder Cancer | ROT |
| Huland | 1990 | Comparison of Different Schedules of Cytostatic Intravesical Instillations in Patients With Superficial Bladder Carcinoma: Final Evaluation of a Prospective Multicenter Study With 419 Patients | ROT |
| DeBruyne | 1988 | BCG (RIVM) Versus Mitomycin Intravesical Therapy in Superficial Bladder Cancer. First Results of Randomized Prospective Trial | ROT |
| Akaza | 1992 | Long-term Results of Intravesical Chemoprophylaxis of Superficial Bladder Cancer: Experience of the Japanese Urological Cancer Research Group for Adriamycin | ROT |
| Witjes | 1993 | A Randomised Prospective Study Comparing Intravesical Instillations of mitomycin-C, BCG-Tice, and BCG-RIVM in pTa-pT1 Tumours and Primary Carcinoma in Situ of the Urinary Bladder. Dutch South-East Cooperative Urological Group | ROT |
| Lundholm | 1996 | A Randomized Prospective Study Comparing Long-Term Intravesical Instillations of Mitomycin C and Bacillus Calmette-Guerin in Patients With Superficial Bladder Carcinoma | ROT |
| Melekos | 1996 | BCG Versus Epirubicin in the Prophylaxis of Multiple Superficial Bladder Tumours: Results of a Prospective Randomized Study Using Modified Treatment Schemes | ROT |
| Cookson | 1997 | The Treated Natural History of High Risk Superficial Bladder Cancer: 15-year Outcome | ROT |
| Herr | 1997 | Tumour Progression and Survival in Patients With T1G3 Bladder Tumours: 15-year Outcome | ROT |
| Tolley | 1988 | Effect of Intravesical Mitomycin C on Recurrence of Newly Diagnosed Superficial Bladder Cancer: Interim Report From the Medical Research Council Subgroup on Superficial Bladder Cancer (Urological Cancer Working Party) | ROT |
| van der Meijden | 2001 | Intravesical Instillation of Epirubicin, Bacillus Calmette-Guerin and Bacillus Calmette-Guerin Plus Isoniazid for Intermediate and High Risk Ta, T1 Papillary Carcinoma of the Bladder: A European Organization for Research and Treatment of Cancer Genito-Urinary Group Randomized Phase III Trial | ROT |
| Oddens | 2014 | The Effect of Age on the Efficacy of Maintenance Bacillus Calmette-Guérin Relative to Maintenance Epirubicin in Patients With Stage Ta T1 Urothelial Bladder Cancer: Results From EORTC Genito-Urinary Group Study 30911 | ROT |
| DeBruyne | 1988 | BCG (RIVM) Versus Mitomycin Intravesical Therapy in Superficial Bladder Cancer. First Results of Randomized Prospective Trial | ROT |
| Witjes | 1996 | Update on the Dutch Cooperative Trial: Mitomycin Versus Bacillus Calmette-Guérin-Tice Versus Bacillus Calmette-Guérin RIVM in the Treatment of Patients With pTA-pT1 Papillary Carcinoma and Carcinoma in Situ of the Urinary Bladder. Dutch South East Cooperative Urological Group | ROT |
| Rintala | 1991 | Intravesical Chemotherapy (Mitomycin C) Versus Immunotherapy (Bacillus Calmette-Guérin) in Superficial Bladder Cancer | ROT |
