# Supplementary material for: Efficacy of intravesical therapies on the prevention of recurrence and progression of non‐muscle‐invasive bladder cancer: A systematic review and network meta‐analysis
Source: Cancer Med. 2020 Oct 11;9(21):7800–9. doi: 10.1002/cam4.3513 (PMC7643689; doi:10.1002/cam4.3513)
Supplement: Supplementary file 4 — File S4 [file CAM4-9-7800-s004.docx]

**Supplementary file 4**

Table S1: Characteristics of included studies and patients

Table S2: Summary of interventions and outcomes in included studies

Table S1: Characteristics of included studies and patients

| Author | Year | Country | Multicenter | Eligible patients | Analyzed patients | Age, year | Male (%) | Tumor | Follow-up |
| --- | --- | --- | --- | --- | --- | --- | --- | --- | --- |
| Brosman et al.[1] | 1982 | America | Single | 47 | 47 | 63.4 | 73.77% | NR | 24-36M |
| Flanigan et al.[2] | 1986 | America | Single | 40 | 40 | NR | NR | Ta/1, Tis | 24M |
| Akaza et al. (First study)[3] | 1987 | Japan | Multicenter | 707 | 575 | Arm 1: 62.3  Arm 2: 62.9  Arm 3: 62.9  Arm 4: 62.9 | 76.87% | Ta/1, G1/2 | 60M |
| Akaza,et al. (Second study)[3] | 1987 | Japan | Multicenter | 665 | 607 | Arm 1: 63.1  Arm 2: 62.1  Arm 3: 62.3  Arm 4: 62.0 | 81.38% | Ta/1, G1/2 | 60M |
| Jauhiainen et al.[4] | 1987 | Finland | Single | 41 | 41 | Arm 1: 68.1(40-82)  Arm 2: 65.2(28-83)  Arm 3: 70.3(58-77) | 84% | Ta/1, G1/2/3 | Arm 1: 23.6m(8-43m);  Arm 2: 23.3m(4-48m);  Arm 3: 14.8m(4-41m). |
| Tsushima et al.[5] | 1987 | Japan | Multicenter | 134 | 103 | 66.1(28-89) | 82.52% | Ta/1, G1/2 | 24M |
| Rubben et al.[6] | 1988 | German | Single | 268 | 220 | Arm 1: 64.2  Arm 2: 64.1  Arm 3: 67.5 | 78.3% | Ta/1, Tis, G1/2/3 | 60M |
| Kim et al.[7] | 1989 | Korea | Single | 43 | 43 | Arm 1: 51.6(36-64)  Arm 2: 57.0(39-71) | 88.37% | High risk, Ta/1, G1/2/3 | 36M |
| Martineiz-Pinero et al.[8] | 1990 | Spain | Single | 202 | 176 | 64(30-87) | 84.66% | Ta/1, Tis, G1/2/3 | Mean 36M |
| Melekos et al.[9] | 1990 | Greece | Single | 100 | 100 | 67.88±8.54 | 85% | Ta/1, G1/2/3 | 24M |
| Pagano et al.[10] | 1991 | Italy | Single | 133 | 133 | 57.4 | 91.00% | Ta/1 | Range 13 to 43 months |
| Lamm et al.[11] | 1991 | America | Multicenter | 285 | 262 | 66.2 | 82.61% | Ta/1, Tis, G1/2/3, recurrent | 60M |
| Hirao et al.[12] | 1992 | Japan | Single | 103 | 93 | Arm 1: 59.1±12.9  Arm 2: 54.2±12.2 | 82.86% | Ta/1, G1/2, primary | Range 1 to 42M |
| Melekos et al.[13] | 1992 | Greece | Single | 80 | 65 | Arm 1: 66.2±17  Arm 2: 67.4±14.3 | 84.62% | Ta/1, Tis, G1/2/3 | Arm 1: Mean 26M  Arm 2: Mean 21M |
| Matsumura et al.[14] | 1992 | Japan | Multicenter | 443 | 284 | ≤49: 22  50-59: 45  60-69: 93  ≥70: 122 | 81.69% | Ta/1, Tis, G1/2/3, recurrent or multiple | 24M |
| Melekos et al.[15] | 1993 | Greece | Multicenter | 190 | 161 | Arm 1: 65.8±8.1  Arm 2: 67.1±12.5  Arm 3: 67.7±5.5 | 83.23% | Ta/1, G1/2/3 | Arm 1: Mean 26M  Arm 2: Mean 28M  Arm 3: Mean 19M |
| Shuin et al.[16] | 1994 | Japan | Multicenter | 68 | 65 | <40: 3  40-49: 4  50-59: 11  60-69: 15  ≥70: 32 | 81.54% | Ta/1, G1/2 | 24M |
| Yasuhiko et al.[17] | 1994 | Japan | Multicenter | 150 | 114 | Arm 1: 68.8(35-88)  Arm 2: 61.9(33-87) | 85.96% | Ta/1, G1/2/3 | Arm 1: 22.5 ± 10.5 M  Arm 2: 20.2 ±10.6 M |
| Obata et al.[18] | 1994 | Japan | Multicenter | 544 | 331 | ≤49: 16  50-59: 33  60-69: 61  ≥70: 56 | 79.52% | Ta/1, G1/2, recurrent or multiple | 53.3M |
| Boccardo et al.[19] | 1994 | America | Multicenter | 287 | 287 | Arm 1: 64(33-82)  Arm 2: 63(20-79) | 86.41% | Ta/1, G1/2, primary | 48M |
| MRC WORKING PARTY ON UROLOGICAL CANCER[20] | 1994 | England | Multicenter | 253 | 246 | 19-51 (14%);  51-59 (23%);  60-69 (37%);  70-79 (24%);  80-89 (2%) | Non-reported | Ta/1, G1/2/3, primary | Median 105M |
| Vegt et al.[21] | 1995 | Netherlands | Multicenter | 469 | 437 | 65.8 | 78.68% | Ta/1, Tis, G1/2/3 | Mean 36M |
| Herr et al.[22] | 1995 | America | Single | 88 | 86 | Arm 1: 61;  Arm 2: 60 | 75.58% | Ta/1, Tis, recurrent | 120M |
| Lamm et al.[23] | 1995 | America | Multicenter | 442 | 377 | Mean range from 66 to 69(28-93) | 83% | Ta/1, Tis, G1/2/3, intermediate to high risk | Median 30.4M |
| Krege et al.[24] | 1996 | New Zealand | Multicenter | 337 | 337 | 65.4±10.4 | 79.82% | Ta/1, G1/2/3 | Median 20.2M |
| Melekos et al.[25] | 1996 | Greece | Single | 132 | 119 | Arm 1: 67.2;  Arm 2: 65.3 | 88.24% | Ta/1, G1/2/3, recurrent or multiple | Mean 35.1M |
| Igawa et al.[26] | 1996 | Japan | Single | 82 | 75 | Arm 1: 66.4  Arm 2: 66.7 | 74.67% | Ta/1, G1/2/3 | 24M |
| Tolley et al.[27] | 1996 | England | Single | 303 | 303 | 24-50: 46  51-60: 115  61-70: 161  71-80: 118  81-100: 12 | Non-reported | Ta/1, G1/2/3, primary | Median 84M |
| Kurth et al.[28] | 1997 | Belgium | Multicenter | 264 | 253 | <50: 19  50-59: 58  60-69: 76  70-79: 87  ≥80: 12  Unknown:1 | 82.68% | Ta/1, Tis, G1/2/3 | Median 40.8M |
| Ali-el-dein et al. a[29] | 1997 | Egypt | Single | 253 | 253 | NR | NR | Ta/1, Tis, G1/2/3, high risk | Mean 30.1M (range from 12 to 48 months) |
| Jimenez-cruz et al.[30] | 1997 | Spain | Multicenter | 122 | 110 | Arm 1: 67.16  Arm 2: 63.75 | 84.43% | T1, G1/2/3, recurrent | 24M |
| Portillo et al.[31] | 1997 | Spain | Single | 90 | 78 | 64.9 | 87.18% | T1, G1/2/3 | 12M |
| Ali-el-dein et al. b[32] | 1997 | Egypt | Single | 181 | 113 | Arm 1: 55(30-68)  Arm 2: 53.4(32-72) | 72.57% | Ta/1, G2/3, high risk | Mean 32.2M |
| Schwaibold et al.[33] | 1997 | German | Multicenter | 597 | 419 | 72(26-91) | 79.5% | Ta/1, Tis, G1/2/3 | Median 57M |
| Giannakopoulos et al.[34] | 1998 | Greece | Single | 89 | 89 | 61.9±9.63 | 80.90% | Ta/1, G2 | 36M |
| Witjes et al.[35] | 1998 | Netherlands | Multicenter | 344 | 327 | <50: 34;  50-59:61;  60-69: 117;  70-79: 105;  ≥80：27 | 81.69% | Ta/1, Tis | Median 86.4M |
| Malmstrom et al.[36] | 1999 | Norway | Multicenter | 261 | 250 | 68 (27-86) | 83.52% | TaG1/2/3, T1G1/2 | Median 64M |
| Malmstrom et al.[37] | 2002 | Sweden | Single | 115 | 110 | 64.9 (37-86) | 85% | Ta/1, G1/2 | NR |
| Stavropoulos et al.[38] | 2002 | Greece | Multicenter | 60 | 54 | Arm 1: 65.7±12.6  Arm 2: 54.3±13.5 | 79.63% | High risk, Ta/1, G2/3 | Mean 12.1M |
| Giannopoulos et al.[39] | 2003 | Greece | Multicenter | 123 | 123 | Arm 1: 68(34,86);  Arm 2: 60(26,81) | 84.55% | Ta/1, G2, primary | Arm 1 median: 26.5M;  Arm 2 median: 24M |
| Cheng et al. a[40] | 2005 | China | Single | 82 | 82 | Arm 1: 65.5  Arm 2: 62.1 | 78.49% | Ta/1, G1/2/3 | Median 131.5M |
| De Reijke et al.[41] | 2005 | Italy | Multicenter | 168 | 102 | <60: 41;  60-69: 55;  70-79: 62;  ≥80: 8  Unknown:2 | 91.67% | Ta/1, Tis | Median 67.2M |
| Cheng et al. b[42] | 2005 | China | Single | 209 | 209 | 69.9(24-92) | 71.29% | Ta/1, G1/2/3 | 144M |
| Liu et al.[43] | 2006 | China | Single | 47 | 47 | 62.1±11.7 | Non-reported | Ta/1, G1/2 | 60M |
| Hinotsu et al.[44] | 2006 | Japan | Multicenter | 83 | 80 | Arm 1: 64.3;  Arm 2: 63.2 | 73.75% | Ta/1, G1/2 | Median 22.2M |
| Gardmark et al.[45] | 2006 | Norway | Multicenter | 261 | 250 | 68(27-86) | 83.52% | TaG1/2/3, T1G1/2 | Median 123M |
| Antonio et al.[46] | 2007 | Spain | Multicenter | 430 | 430 | 64.5 | 87.00% | Intermediate risk, Ta/1, G1/2 | Median 52.6M |
| Friedrich et al.[47] | 2007 | German | Multicenter | 495 | 495 | Arm 1: 67(36-85)  Arm 2: 68(38-89)  Arm 3: 67(36-84) | 80.20% | Intermediate or high risk | Median 34.8M |
| Mangiarotti et al.[48] | 2008 | Italy | Single | 92 | 92 | 64.0±12.3 | 72.83% | Ta/1, G1/2 | Mean 65.7±25.6M |
| Jarvinen et al.[49] | 2009 | Finland | Multicenter | 89 | 89 | Arm 1: 68(50-85)  Arm 2: 67(31-84) | 71.91% | Ta/1, G1/2/3, recurrent | Median 102M |
| Porena et al.[50] | 2010 | Italy | Single | 64 | 64 | Arm 1: 68.7±10.2  Arm 2: 70.2±5.5 | 84.38% | High risk | Mean 44M |
| Lorenzo et al.[51] | 2010 | Italy | Multicenter | 80 | 80 | Arm 1: 69.3±8.4  Arm 2: 71.4±7.9 | 61.25% | High risk, BCG failure | Median 19M |
| Sylvester et al.[52] | 2010 | Netherlands | Multicenter | 957 | 837 | 67(IQR60-72) | 77% | Intermediate to high risk | Median 110.4M |
| Hinotsu et al.[53] | 2010 | Japan | Multicenter | 120 | 116 | ≤64: 50  ≥65: 65 | 90.43% | Ta/1, recurrent or multiple | Median 24M |
| Addeo et al.[54] | 2010 | Italy | Multicenter | 109 | 109 | Arm 1: 67.9  Arm 2: 64.9 | 85.32% | Ta/1, G1/2/3, recurrent | Median 36M |
| Gontero et al.[55] | 2013 | Italy | Multicenter | 88 | 88 | 67.4±9.5 (37-84) | 85.83% | Intermediate risk | 12M |
| Mondal et al.[56] | 2016 | India | Single | 40 | 40 | 31-41: 5  41-50: 6  51-60: 11  61-70: 11  71-80: 6  81-90: 1 | 85% | Ta/1, G1/2 | 6M |

Table S2: Summary of interventions and outcomes in included studies

| Study number | Arm | Intervention | Dose (drug/normal saline) | Number of instillations | Schedule | Recurrence | | Progression | |
| --- | --- | --- | --- | --- | --- | --- | --- | --- | --- |
|  |  |  |  |  |  | lnHR | se(lnHR) | lnHR | se(lnHR) |
| Brosman et al.[1] | 1 | BCG (Tice) | 6×10^9^ CFU/60cc | 30-33 | M | - | - | - | - |
|  | 2 | THP | 60mg/60cc | 30-33 | M | - | - | - | - |
| Flanigan et al.[2] | 1 | MMC | 40mg/40ml | 30 | M | 0.18 | 0.84 | 0.53 | 1.03 |
|  | 2 | THP | 60mg/60ml | 30 | M | Ref. | Ref. | Ref. | Ref. |
| Akaza et al. (First study)[3] | 1 | ADM | 30mg/30ml | 8 | I | -0.3 | 0.16 | - | - |
|  | 2 | ADM | 20mg/40ml | 8 | I | -0.5 | 0.17 | - | - |
|  | 3 | MMC | 20mg/40ml | 8 | I | -0.36 | 0.17 | - | - |
|  | 4 | TURBT | - | - | - | Ref. | Ref. | - | - |
| Akaza,et al. (Second study)[3] | 1 | ADM | 30mg/30ml | 21 | M | -0.29 | 0.18 | - | - |
|  | 2 | ADM | 20mg/40ml | 21 | M | -0.34 | 0.18 | - | - |
|  | 3 | MMC | 20mg/40ml | 21 | M | -0.35 | 0.18 | - | - |
|  | 4 | TURBT | - | - | - | Ref. | Ref. | - | - |
| Jauhiainen et al.[4] | 1 | MMC | 20-40mg/phosphate buffer | repeated 5 times weekly and thereafter monthly | M | -1.63 | 0.61 |  |  |
|  | 2 | ADM | 20-40mg/phosphate buffer | repeated 5 times weekly and thereafter monthly | M | Ref. | Ref. |  |  |
| Tsushima et al.[5] | 1 | ADM | 50mg/100ml | 52-54 | M | -1.25 | 0.4 |  |  |
|  | 2 | MMC | 30mg/100ml | 52-54 | M | -0.59 | 0.26 |  |  |
|  | 3 | TURBT | - | - | - | Ref. | Ref. |  |  |
| Rubben et al.[6] | 1 | ADM | 50mg/50ml | 12 | I | -0.1 | 0.13 | 0.29 | 0.38 |
|  | 2 | ADM | 50mg/50ml | 27 | M | -0.07 | 0.13 | -0.11 | 0.43 |
|  | 3 | TURBT | - | - | - | Ref. | Ref. | Ref. | Ref. |
| Kim et al.[7] | 1 | MMC | 40mg/40ml | 8 | I | 0.06 | 0.26 | -0.65 | 0.82 |
|  | 2 | TURBT | - | - | - | Ref. | Ref. | Ref. | Ref. |
| Martineiz-Pinero et al.[8] | 1 | ADM | 50mg/50ml | 15 | M | 0.19 | 0.24 | 0.75 | 0.86 |
|  | 2 | BCG (Pasteur) | 150mg/50ml | 15 | M | -1.17 | 0.4 | -0.87 | 1.23 |
|  | 3 | THP | 50mg/50ml | 15 | M | Ref. | Ref. | Ref. | Ref. |
| Melekos et al.[9] | 1 | BCG (Pasteur) | 150mg/50ml | 8 | I | -0.56 | 0.23 | -1.33 | 0.42 |
|  | 2 | TURBT | - | - | - | Ref. | Ref. | Ref. | Ref. |
| Pagano et al.[10] | 1 | BCG (Pasteur) | 75mg/50ml | 22 | M | -1.09 | 0.2 | -1.4 | 0.64 |
|  | 2 | TURBT | - | - | - | Ref. | Ref. | Ref. | Ref. |
| Lamm et al.[11] | 1 | BCG (Connaught) | 150mg/50.5ml | 11 | M | -0.34 | 0.08 | - | - |
|  | 2 | ADM | 50mg/50ml | 16 | M | Ref. | Ref. | - | - |
| Hirao et al.[12] | 1 | THP | 30mg/30ml | 32 | M | -1.12 | 0.37 | - | - |
|  | 2 | TURBT | - | - | - | Ref. | Ref. | - | - |
| Melekos et al.[13] | 1 | EPI | 50mg/5ml | 16 | M | -0.64 | 0.4 | -0.67 | 0.98 |
|  | 2 | TURBT | - | - | - | Ref. | Ref. | Ref. | Ref. |
| Matsumura et al.[14] | 1 | ADM | 20mg/40ml | 21 | M | -0.24 | 0.08 | - | - |
|  | 2 | ADM | 20mg/40ml | 6 | I | 0.04 | 0.06 | - | - |
|  | 3 | TURBT | - | - | - | Ref. | Ref. | - | - |
| Melekos et al.[15] | 1 | EPI | 50mg/50ml | >16 | M | -0.62 | 0.32 | -0.89 | 0.52 |
|  | 2 | BCG (Pasteur) | 150mg/50ml | >14 | M | -0.95 | 0.34 | -1.22 | 0.6 |
|  | 3 | TURBT | - | - | - | Ref. | Ref. | Ref. | Ref. |
| Shuin et al.[16] | 1 | EPI | 30mg/40ml | 18 | M | -0.09 | 0.42 | - | - |
|  | 2 | ADM | 30mg/40ml | 18 | M | Ref. | Ref. | - | - |
| Yasuhiko et al.[17] | 1 | EPI | 30mg/30ml | 19 | M | -0.51 | 0.49 | -1.2 | 1.14 |
|  | 2 | ADM | 30mg/30ml | 19 | M | Ref. | Ref. | Ref. | Ref. |
| Obata et al.[18] | 1 | ADM | 20mg/40ml | 19 | M | -1.24 | 0.33 | - | - |
|  | 2 | TURBT | - | - | - | Ref. | Ref. | - | - |
| Boccardo et al.[19] | 1 | MMC | 40mg/50ml | 8 | I | -0.38 | 0.18 | - | - |
|  | 2 | IFNα-2b | 50×10^6^ IU/50ml | 8 | I | Ref. | Ref. | - | - |
| MRC [20] | 1 | THP | 30mg/50ml | 5 | I | 0.08 | 0.18 | - | - |
|  | 2 | TURBT | - | - | - | Ref. | Ref. | - | - |
| Vegt et al.[21] | 1 | BCG (TICE) | 5 x 10^8/50ml | 6 | I | 0.45 | 0.17 | 0.02 | 0.5 |
|  | 2 | BCG (RIVM) | 5 x 10^8/50ml | 6 | I | 0.11 | 0.18 | 0.01 | 0.48 |
|  | 3 | MMC | 30ml/50ml | 10 | M | Ref. | Ref. | Ref. | Ref. |
| Herr et al.[22] | 1 | BCG (Armand Frappier, Montreal, Canada) | 120mg/50ml | 6 | I | - | - | -0.84 | 0.31 |
|  | 2 | TURBT | - | - | - | - | - | Ref. | Ref. |
| Lamm et al.[23] | 1 | BCG (Tice) | 5×10^8^/50ml | 17 | M | -0.36 | 0.15 | -0.23 | 0.32 |
|  | 2 | MMC | 20mg/20ml | 17 | M | Ref. | Ref. | Ref. | Ref. |
| Krege et al.[24] | 1 | MMC | 20mg/50ml | 36 | M | -0.68 | 0.23 | - | - |
|  | 2 | BCG (Connaught) | 120mg/50ml | 10 | I | -0.48 | 0.24 | - | - |
|  | 3 | TURBT | - | - | - | Ref. | Ref. | - | - |
| Melekos et al.[25] | 1 | BCG (Tice) | 5×10^8^/50ml | 14 or 16 | M | -0.2 | 0.27 | 0.31 | 0.46 |
|  | 2 | EPI | 50mg/50ml | 12 or 16 | M | Ref. | Ref. | Ref. | Ref. |
| Igawa et al.[26] | 1 | EPI | 20mg/40ml | 24 | M | -0.12 | 0.29 | 1.9 | 1.03 |
|  | 2 | TURBT | - | - | - | Ref. | Ref. | Ref. | Ref. |
| Tolley et al.[27] | 1 | MMC | 40mg/40ml | 5 | I | -0.69 | 0.17 | -2 | 0.37 |
|  | 2 | TURBT | - | - | - | Ref. | Ref. | Ref. | Ref. |
| Kurth et al.[28] | 1 | ADM | 50mg/50ml | 15 | M | -0.41 | 0.19 | -0.26 | 0.37 |
|  | 2 | TURBT | - | - | - | Ref. | Ref. | Ref. | Ref. |
| Ali-el-dein et al.[29] | 1 | EPI | 50mg/50ml | 18 | M | -0.93 | 0.27 | 0.29 | 0.57 |
|  | 2 | EPI | 80mg/50ml | 18 | M | -1.06 | 0.28 | -0.62 | 0.72 |
|  | 3 | ADM | 50mg/50ml | 18 | M | -0.55 | 0.25 | 0.2 | 0.59 |
|  | 4 | TURBT | - | - | - | Ref. | Ref. | Ref. | Ref. |
| Jimenez-cruz et al.[30] | 1 | BCG (Pasteur) | 150mg/50ml | 17 | M | -0.57 | 0.21 | -0.37 | 0.53 |
|  | 2 | Recombinant IFNα-2a | 54MU/50ml | 17 | M | Ref. | Ref. | Ref. | Ref. |
| Portillo et al.[31] | 1 | IFNα-2b | 60million units | 24 | M | 0 | 0.31 | -0.01 | 1.04 |
|  | 2 | TURBT | - | - | - | Ref. | Ref. | Ref. | Ref. |
| Ali-el-dein et al.[32] | 1 | EPI | 50mg/50ml | 18 | M | -0.71 | 0.26 | -1 | 0.82 |
|  | 2 | TURBT | - | - | - | Ref. | Ref. | Ref. | Ref. |
| Schwaibold et al.[33] | 1 | MMC | 20mg/20ml | 42 | M | -0.23 | 0.27 | -0.45 | 0.39 |
|  | 2 | MMC | 20mg/20ml | 42 | M | -0.55 | 0.33 | -1.24 | 0.55 |
|  | 3 | MMC | 20mg/20ml | 20 | M | -0.43 | 0.33 | -0.99 | 0.55 |
|  | 4 | ADM | 50mg/50ml | 42 | M | Ref. | Ref. | Ref. | Ref. |
| Giannakopoulos et al.[34] | 1 | IFNα-2b | 40MU/50ml | 22 | M | -0.58 | 0.33 | -0.79 | 0.65 |
|  | 2 | IFNα-2b | 60MU/50ml | 22 | M | -0.8 | 0.36 | -1.97 | 1.05 |
|  | 3 | IFNα-2b | 80MU/50ml | 22 | M | -1.1 | 0.43 | -1.93 | 1.03 |
|  | 4 | TURBT | - | - | - | Ref. | Ref. | Ref. | Ref. |
| Witjes et al.[35] | 1 | BCG (RIVM) | 5×10^8^/50ml | 6 | I | 0.15 | 0.16 | 0.59 | 0.35 |
|  | 2 | MMC | 30mg/50ml | 10 | I | Ref. | Ref. | Ref. | Ref. |
| Malmstrom et al. [36] | 1 | MMC | 40mg/50ml | 20-21 | M | -0.34 | 0.16 | - | - |
|  | 2 | BCG (Danish) | 120mg/50ml | 20-21 | M | Ref. | Ref. | - | - |
| Malmstrom et al.[37] | 1 | IFN-α | 30MU/30ml | 12 | I | 1.68 | 0.6 | - | - |
|  | 2 | IFN-α | 50MU/30ml | 12 | I | 1.46 | 0.6 | - | - |
|  | 3 | IFN-α | 80MU/30ml | 12 | I | 1.17 | 0.64 | - | - |
|  | 4 | MMC | 40mg/40ml | 8 | I | Ref. | Ref. | - | - |
| Stavropoulos et al.[38] | 1 | IFN γ | 0.7mg/50ml | 8 | I | -1.17 | 0.52 | 0.07 | 1.41 |
|  | 2 | TURBT | - | - | - | Ref. | Ref. | Ref. | Ref. |
| Giannopoulos et al.[39] | 1 | MMC | 40mg/50ml | 20 | M | -0.6 | 0.31 | 0.64 | 0.86 |
|  | 2 | IFN γ-1b | 0.5mg/50ml | 20 | M | Ref. | Ref. | Ref. | Ref. |
| Cheng et al.[40] | 1 | ADM | 50mg/50ml | 11 | M | -0.52 | 0.33 | 0.51 | 0.67 |
|  | 2 | TURBT | - | - | - | Ref. | Ref. | Ref. | Ref. |
| De Reijke et al.[41] | 1 | BCG (Connaught) | 81mg/np | 27 | M | -1.01 | 0.29 | -0.45 | 0.32 |
|  | 2 | EPI | 50mg/np | 29 | M | Ref. | Ref. | Ref. | Ref. |
| Cheng t al.[42] | 1 | BCG (Connaught) | 81mg/50ml | 16 | M | -0.75 | 0.21 | -0.15 | 0.35 |
|  | 2 | EPI | 50mg/50ml | 11 | M | Ref. | Ref. | Ref. | Ref. |
| Liu et al.[43] | 1 | MMC | 40mg/40ml | 16-18 | M | -0.13 | 1.4 | - | - |
|  | 2 | EPI | 40mg/40ml | 16-18 | M | Ref. | Ref. | - | - |
| Hinotsu et al.[44] | 1 | BCG (Tokyo 172) | 80mg/40ml | 6 | I | -0.68 | 0.33 | - | - |
|  | 2 | ADM | 20mg/40ml | 17 | M | Ref. | Ref. | - | - |
| Gardmark et al.[45] | 1 | BCG (Danish) | 120mg/50ml | 20-21 | M | - | - | -0.3 | 0.27 |
|  | 2 | MMC | 40mg/50ml | 20-21 | M | - | - | Ref. | Ref. |
| Antonio et al.[46] | 1 | BCG | 27mg/np | 12 | M | -0.44 | 0.2 | 0.02 | 0.38 |
|  | 2 | BCG | 13.5mg/np | 12 | M | -0.13 | 0.19 | 0.27 | 0.34 |
|  | 3 | MMC | 30mg/np | 12 | M | Ref. | Ref. | Ref. | Ref. |
| Friedrich et al.[47] | 1 | MMC | 20mg/20ml | 6 | I | -0.87 | 0.26 | - | - |
|  | 2 | MMC | 20mg/20ml | 42 | M | -0.09 | 0.25 | - | - |
|  | 3 | BCG (RIVM) | 2×10^8^cfu/20ml | 6 | I | Ref. | Ref. | - |  |
| Mangiarotti et al.[48] | 1 | BCG (Tice) | NR | 18 | M | 0.17 | 0.29 | - | - |
|  | 2 | MMC | 40mg/50ml | 20 | M | Ref. | Ref. | - | - |
| Jarvinen et al.[49] | 1 | BCG (Pasteur) | 75mg/50ml | 27 | M | -0.71 | 0.25 | -0.89 | 0.56 |
|  | 2 | MMC | 30-40mg/150-200ml phosphate buffer | 27 | M | Ref. | Ref. | Ref. | Ref. |
| Porena et al.[50] | 1 | BCG (Tice) | 5×10^8^ CFU/50ml | 13 | M | -0.8 | 0.39 | - | - |
|  | 2 | GEM | 2000mg/50ml | 13 | M | Ref. | Ref. | - | - |
| Lorenzo et al.[51] | 1 | BCG (Connaught) | 81mg/50ml | 21 | M | 0.89 | 0.25 | 0.12 | 0.46 |
|  | 2 | GEM | 2000mg/50ml | 21 | M | Ref. | Ref. | Ref. | Ref. |
| Sylvester et al.[52] | 1 | BCG (Tice) | 5×10^8^cfu/np | 27 | M | -0.48 | 0.11 | -0.17 | 0.26 |
|  | 2 | EPI | 50mg/50ml | 27 | M | Ref. | Ref. | Ref. | Ref. |
| Hinotsu et al.[53] | 1 | BCG (Connaught) | 81mg/40ml | 18 | M | -1.77 | 0.41 | - | - |
|  | 2 | BCG (Connaught) | 81mg/40ml | 6 | I | -0.77 | 0.35 | -1.12 | 0.66 |
|  | 3 | EPI | 40mg/40ml | 9 | Unclear | Ref. | Ref. | Ref. | Ref. |
| Addeo et al.[54] | 1 | MMC | 40mg/50ml | 15 | M | 0.64 | 0.28 | 0.41 | 0.26 |
|  | 2 | GEM | 2000mg/50ml | 16 | M | Ref. | Ref. | Ref. | Ref. |
| Gontero et al.[55] | 1 | BCG (Connaught) | 27mg/50ml | 15 | M | -0.08 | 0.37 | -0.27 | 0.73 |
|  | 2 | GEM | 2000mg/50ml | 17 | M | Ref. | Ref. | Ref. | Ref. |
| Mondal et al.[56] | 1 | MMC | 40mg/50ml | 6 | I | - | - | - | - |
|  | 2 | BCG (Danish) | 120mg/50ml | 6 | I | - | - | - | - |

**Reference**

[1] Brosman SA. Experience with bacillus Calmette-Guerin in patients with superficial bladder carcinoma. The Journal of urology. 1982;128:27-30.

[2] Flanigan RC, Ellison MF, Butler KM, Gomella LG, McRoberts JW. A trial of prophylactic thiotepa or mitomycin C intravesical therapy in patients with recurrent or multiple superficial bladder cancers. The Journal of urology. 1986;136:35-7.

[3] Akaza H, Isaka S, Koiso K, Kotake T, Machida T, Maru A, et al. Comparative analysis of short-term and long-term prophylactic intravesical chemotherapy of superficial bladder cancer. Prospective, randomized, controlled studies of the Japanese Urological Cancer Research Group. Cancer chemotherapy and pharmacology. 1987;20 Suppl:S91-6.

[4] Jauhiainen K, Alfthan O. Instillation of mitomycin C and doxorubicin in the prevention of recurrent superficial (Ta-T1) bladder cancer. British journal of urology. 1987;60:54-9.

[5] Tsushima T, Matsumura Y, Ozaki Y, Yoshimoto J, Ohmori H. Prophylactic intravesical instillation therapy with adriamycin and mitomycin C in patients with superficial bladder cancer. Cancer chemotherapy and pharmacology. 1987;20 Suppl:S72-6.

[6] Rübben H, Lutzeyer W, Fischer N, Deutz F, Lagrange W, Giani G. Natural history and treatment of low and high risk superficial bladder tumors. The Journal of urology. 1988;139:283-5.

[7] Kim HH, Lee C. Intravesical mitomycin C instillation as a prophylactic treatment of superficial bladder tumor. The Journal of urology. 1989;141:1337-9; discussion 9-40.

[8] Martínez-Piñeiro JA, Jiménez León J, Martínez-Piñeiro L, Jr., Fiter L, Mosteiro JA, Navarro J, et al. Bacillus Calmette-Guerin versus doxorubicin versus thiotepa: a randomized prospective study in 202 patients with superficial bladder cancer. The Journal of urology. 1990;143:502-6.

[9] Melekos MD. Intravesical Bacillus Calmette-Guérin prophylactic treatment for superficial bladder tumors: results of a controlled prospective study. Urologia internationalis. 1990;45:137-41.

[10] Pagano F, Bassi P, Milani C, Meneghini A, Maruzzi D, Garbeglio A. A low dose bacillus Calmette-Guerin regimen in superficial bladder cancer therapy: is it effective? The Journal of urology. 1991;146:32-5.

[11] Lamm DL, Blumenstein BA, Crawford ED, Montie JE, Scardino P, Grossman HB, et al. A randomized trial of intravesical doxorubicin and immunotherapy with bacille Calmette-Guérin for transitional-cell carcinoma of the bladder. The New England journal of medicine. 1991;325:1205-9.

[12] Hirao Y, Okajima E, Ozono S, Samma S, Sasaki K, Hiramatsu T, et al. A prospective randomized study of prophylaxis of tumor recurrence following transurethral resection of superficial bladder cancer--intravesical thio-TEPA versus oral UFT. Cancer chemotherapy and pharmacology. 1992;30 Suppl:S26-30.

[13] Melekos MD, Dauaher H, Fokaefs E, Barbalias G. Intravesical instillations of 4-epi-doxorubicin (epirubicin) in the prophylactic treatment of superficial bladder cancer: results of a controlled prospective study. The Journal of urology. 1992;147:371-5.

[14] Matsumura Y, Akaza H, Isaka S, Kagawa S, Koiso K, Kotake T, et al. The 4th study of prophylactic intravesical chemotherapy with adriamycin in the treatment of superficial bladder cancer: the experience of the Japanese Urological Cancer Research Group for Adriamycin. Cancer chemotherapy and pharmacology. 1992;30 Suppl:S10-4.

[15] Melekos MD, Chionis HS, Paranychianakis GS, Dauaher HH. Intravesical 4'-epi-doxorubicin (epirubicin) versus bacillus Calmette-Guérin. A controlled prospective study on the prophylaxis of superficial bladder cancer. Cancer. 1993;72:1749-55.

[16] Shuin T, Kubota Y, Noguchi S, Hosaka M, Miura T, Kondo I, et al. A phase II study of prophylactic intravesical chemotherapy with 4'-epirubicin in recurrent superficial bladder cancer: comparison of 4'-epirubicin and adriamycin. Cancer chemotherapy and pharmacology. 1994;35 Suppl:S52-6.

[17] Eto H, Oka Y, Ueno K, Nakamura I, Yoshimura K, Arakawa S, et al. Comparison of the prophylactic usefulness of epirubicin and doxorubicin in the treatment of superficial bladder cancer by intravesical instillation: a multicenter randomized trial. Kobe University Urological Oncology Group. Cancer chemotherapy and pharmacology. 1994;35 Suppl:S46-51.

[18] Obata K, Ohashi Y, Akaza H, Isaka S, Kagawa S, Koiso K, et al. Prophylactic chemotherapy with intravesical instillation of adriamycin and oral administration of 5-fluorouracil after surgery for superficial bladder cancer. The Japanese Urological Cancer Research Group for Adriamycin. Cancer chemotherapy and pharmacology. 1994;35 Suppl:S88-92.

[19] Boccardo F, Cannata D, Rubagotti A, Guarneri D, Decensi A, Canobbio L, et al. Prophylaxis of superficial bladder cancer with mitomycin or interferon alfa-2b: results of a multicentric Italian study. Journal of clinical oncology : official journal of the American Society of Clinical Oncology. 1994;12:7-13.

[20] MRC. The effect of intravesical thiotepa on tumour recurrence after endoscopic treatment of newly diagnosed superficial bladder cancer. A further report with long-term follow-up of a Medical Research Council randomized trial. Medical Research Council Working Party on Urological Cancer, Subgroup on Superficial Bladder Cancer. British journal of urology. 1994;73:632-8.

[21] Vegt PD, Witjes JA, Witjes WP, Doesburg WH, Debruyne FM, van der Meijden AP. A randomized study of intravesical mitomycin C, bacillus Calmette-Guerin Tice and bacillus Calmette-Guerin RIVM treatment in pTa-pT1 papillary carcinoma and carcinoma in situ of the bladder. The Journal of urology. 1995;153:929-33.

[22] Herr HW, Schwalb DM, Zhang ZF, Sogani PC, Fair WR, Whitmore WF, Jr., et al. Intravesical bacillus Calmette-Guérin therapy prevents tumor progression and death from superficial bladder cancer: ten-year follow-up of a prospective randomized trial. Journal of clinical oncology : official journal of the American Society of Clinical Oncology. 1995;13:1404-8.

[23] Lamm DL, Blumenstein BA, David Crawford E, Crissman JD, Lowe BA, Smith JA, Jr., et al. Randomized intergroup comparison of bacillus calmette-guerin immunotherapy and mitomycin C chemotherapy prophylaxis in superficial transitional cell carcinoma of the bladder a southwest oncology group study. Urologic oncology. 1995;1:119-26.

[24] Krege S, Giani G, Meyer R, Otto T, Rübben H. A randomized multicenter trial of adjuvant therapy in superficial bladder cancer: transurethral resection only versus transurethral resection plus mitomycin C versus transurethral resection plus bacillus Calmette-Guerin. Participating Clinics. The Journal of urology. 1996;156:962-6.

[25] Melekos MD, Zarakovitis IE, Fokaefs ED, Dandinis K, Chionis H, Bouropoulos C, et al. Intravesical bacillus Calmette-Guérin versus epirubicin in the prophylaxis of recurrent and/or multiple superficial bladder tumours. Oncology. 1996;53:281-8.

[26] Igawa M, Urakami S, Shirakawa H, Shiina H, Ishibe T, Kadena H, et al. Intravesical instillation of epirubicin: effect on tumour recurrence in patients with dysplastic epithelium after transurethral resection of superficial bladder tumour. British journal of urology. 1996;77:358-62.

[27] Tolley DA, Parmar MK, Grigor KM, Lallemand G, Benyon LL, Fellows J, et al. The effect of intravesical mitomycin C on recurrence of newly diagnosed superficial bladder cancer: a further report with 7 years of follow up. The Journal of urology. 1996;155:1233-8.

[28] Kurth K, Tunn U, Ay R, Schröder FH, Pavone-Macaluso M, Debruyne F, et al. Adjuvant chemotherapy for superficial transitional cell bladder carcinoma: long-term results of a European Organization for Research and Treatment of Cancer randomized trial comparing doxorubicin, ethoglucid and transurethral resection alone. The Journal of urology. 1997;158:378-84.

[29] Ali-el-Dein B, el-Baz M, Aly AN, Shamaa S, Ashamallah A. Intravesical epirubicin versus doxorubicin for superficial bladder tumors (stages pTa and pT1): a randomized prospective study. The Journal of urology. 1997;158:68-73; discussion -4.

[30] Jimenez-Cruz JF, Vera-Donoso CD, Leiva O, Pamplona M, Rioja-Sanz LA, Martinez-Lasierra M, et al. Intravesical immunoprophylaxis in recurrent superficial bladder cancer (Stage T1): multicenter trial comparing bacille Calmette-Guérin and interferon-alpha. Urology. 1997;50:529-35.

[31] Portillo J, Martin B, Hernandez R, Correas M, Gutierrez J, Del Valle J, et al. Results at 43 months' follow-up of a double-blind, randomized, prospective clinical trial using intravesical interferon alpha-2b in the prophylaxis of stage pT1 transitional cell carcinoma of the bladder. Urology. 1997;49:187-90.

[32] Ali-el-Dein B, Nabeeh A, el-Baz M, Shamaa S, Ashamallah A. Single-dose versus multiple instillations of epirubicin as prophylaxis for recurrence after transurethral resection of pTa and pT1 transitional-cell bladder tumours: a prospective, randomized controlled study. British journal of urology. 1997;79:731-5.

[33] Schwaibold H, Pichlmeier U, Klingenberger HJ, Huland H. Long-term follow-up of cytostatic intravesical instillation in patients with superficial bladder carcinoma. Is short-term, intensive instillation better than maintenance therapy? European urology. 1997;31:153-9.

[34] Giannakopoulos S, Gekas A, Alivizatos G, Sofras F, Becopoulos T, Dimopoulos C. Efficacy of escalating doses of intravesical interferon alpha-2b in reducing recurrence rate and progression in superficial transitional cell carcinoma. British journal of urology. 1998;82:829-34.

[35] Witjes JA, v d Meijden AP, Collette L, Sylvester R, Debruyne FM, van Aubel A, et al. Long-term follow-up of an EORTC randomized prospective trial comparing intravesical bacille Calmette-Guérin-RIVM and mitomycin C in superficial bladder cancer. EORTC GU Group and the Dutch South East Cooperative Urological Group. European Organisation for Research and Treatment of Cancer Genito-Urinary Tract Cancer Collaborative Group. Urology. 1998;52:403-10.

[36] Malmström PU, Wijkström H, Lundholm C, Wester K, Busch C, Norlén BJ. 5-year followup of a randomized prospective study comparing mitomycin C and bacillus Calmette-Guerin in patients with superficial bladder carcinoma. Swedish-Norwegian Bladder Cancer Study Group. The Journal of urology. 1999;161:1124-7.

[37] Malmström PU. A randomized comparative dose-ranging study of interferon-alpha and mitomycin-C as an internal control in primary or recurrent superficial transitional cell carcinoma of the bladder. BJU international. 2002;89:681-6.

[38] Stavropoulos NE, Hastazeris K, Filiadis I, Mihailidis I, Ioachim E, Liamis Z, et al. Intravesical instillations of interferon gamma in the prophylaxis of high risk superficial bladder cancer--results of a controlled prospective study. Scandinavian journal of urology and nephrology. 2002;36:218-22.

[39] Giannopoulos A, Constantinides C, Fokaeas E, Stravodimos C, Giannopoulou M, Kyroudi A, et al. The immunomodulating effect of interferon-gamma intravesical instillations in preventing bladder cancer recurrence. Clinical cancer research : an official journal of the American Association for Cancer Research. 2003;9:5550-8.

[40] Cheng CW, Chan PS, Chan LW, Chan CK, Ng CF, Lai MM. 17-year follow-up of a randomized prospective controlled trial of adjuvant intravesical doxorubicin in the treatment of superficial bladder cancer. International braz j urol : official journal of the Brazilian Society of Urology. 2005;31:204-11.

[41] de Reijke TM, Kurth KH, Sylvester RJ, Hall RR, Brausi M, van de Beek K, et al. Bacillus Calmette-Guerin versus epirubicin for primary, secondary or concurrent carcinoma in situ of the bladder: results of a European Organization for the Research and Treatment of Cancer--Genito-Urinary Group Phase III Trial (30906). The Journal of urology. 2005;173:405-9.

[42] Cheng CW, Chan SF, Chan LW, Chan CK, Ng CF, Cheung HY, et al. Twelve-year follow up of a randomized prospective trial comparing bacillus Calmette-Guerin and epirubicin as adjuvant therapy in superficial bladder cancer. International journal of urology : official journal of the Japanese Urological Association. 2005;12:449-55.

[43] Liu B, Wang Z, Chen B, Yu J, Zhang P, Ding Q, et al. Randomized study of single instillation of epirubicin for superficial bladder carcinoma: long-term clinical outcomes. Cancer investigation. 2006;24:160-3.

[44] Hinotsu S, Akaza H, Isaka S, Kanetake H, Kubota Y, Kuroda M, et al. Sustained prophylactic effect of intravesical bacille Calmette-Guérin for superficial bladder cancer: a smoothed hazard analysis in a randomized prospective study. Urology. 2006;67:545-9.

[45] Gårdmark T, Jahnson S, Wahlquist R, Wijkström H, Malmström PU. Analysis of progression and survival after 10 years of a randomized prospective study comparing mitomycin-C and bacillus Calmette-Guérin in patients with high-risk bladder cancer. BJU international. 2007;99:817-20.

[46] Ojea A, Nogueira JL, Solsona E, Flores N, Gómez JM, Molina JR, et al. A multicentre, randomised prospective trial comparing three intravesical adjuvant therapies for intermediate-risk superficial bladder cancer: low-dose bacillus Calmette-Guerin (27 mg) versus very low-dose bacillus Calmette-Guerin (13.5 mg) versus mitomycin C. European urology. 2007;52:1398-406.

[47] Friedrich MG, Pichlmeier U, Schwaibold H, Conrad S, Huland H. Long-term intravesical adjuvant chemotherapy further reduces recurrence rate compared with short-term intravesical chemotherapy and short-term therapy with Bacillus Calmette-Guérin (BCG) in patients with non-muscle-invasive bladder carcinoma. European urology. 2007;52:1123-29.

[48] Mangiarotti B, Trinchieri A, Del Nero A, Montanari E. A randomized prospective study of intravesical prophylaxis in non-musle invasive bladder cancer at intermediate risk of recurrence: mitomycin chemotherapy vs BCG immunotherapy. Archivio italiano di urologia, andrologia : organo ufficiale [di] Societa italiana di ecografia urologica e nefrologica. 2008;80:167-71.

[49] Järvinen R, Kaasinen E, Sankila A, Rintala E. Long-term efficacy of maintenance bacillus Calmette-Guérin versus maintenance mitomycin C instillation therapy in frequently recurrent TaT1 tumours without carcinoma in situ: a subgroup analysis of the prospective, randomised FinnBladder I study with a 20-year follow-up. European urology. 2009;56:260-5.

[50] Porena M, Del Zingaro M, Lazzeri M, Mearini L, Giannantoni A, Bini V, et al. Bacillus Calmette-Guérin versus gemcitabine for intravesical therapy in high-risk superficial bladder cancer: a randomised prospective study. Urologia internationalis. 2010;84:23-7.

[51] Di Lorenzo G, Perdonà S, Damiano R, Faiella A, Cantiello F, Pignata S, et al. Gemcitabine versus bacille Calmette-Guérin after initial bacille Calmette-Guérin failure in non-muscle-invasive bladder cancer: a multicenter prospective randomized trial. Cancer. 2010;116:1893-900.

[52] Sylvester RJ, Brausi MA, Kirkels WJ, Hoeltl W, Calais Da Silva F, Powell PH, et al. Long-term efficacy results of EORTC genito-urinary group randomized phase 3 study 30911 comparing intravesical instillations of epirubicin, bacillus Calmette-Guérin, and bacillus Calmette-Guérin plus isoniazid in patients with intermediate- and high-risk stage Ta T1 urothelial carcinoma of the bladder. European urology. 2010;57:766-73.

[53] Hinotsu S, Akaza H, Naito S, Ozono S, Sumiyoshi Y, Noguchi S, et al. Maintenance therapy with bacillus Calmette-Guérin Connaught strain clearly prolongs recurrence-free survival following transurethral resection of bladder tumour for non-muscle-invasive bladder cancer. BJU international. 2011;108:187-95.

[54] Addeo R, Caraglia M, Bellini S, Abbruzzese A, Vincenzi B, Montella L, et al. Randomized phase III trial on gemcitabine versus mytomicin in recurrent superficial bladder cancer: evaluation of efficacy and tolerance. Journal of clinical oncology : official journal of the American Society of Clinical Oncology. 2010;28:543-8.

[55] Gontero P, Oderda M, Mehnert A, Gurioli A, Marson F, Lucca I, et al. The impact of intravesical gemcitabine and 1/3 dose Bacillus Calmette-Guérin instillation therapy on the quality of life in patients with nonmuscle invasive bladder cancer: results of a prospective, randomized, phase II trial. The Journal of urology. 2013;190:857-62.

[56] Mondal H, Yirang K, Mukhopadhyay C, Adhikary S, Dutta B, Bhoj S. Prospective Randomized Study between Intravesical BCG and Mitomycin-C for Non-Muscle-Invasive Urothelial Carcinoma of Urinary-Bladder Post TURBT. Bangladesh Journal of Medical Science 2016;15:74-7.
