# Supplementary material for: Efficacy of intravesical therapies on the prevention of recurrence and progression of non‐muscle‐invasive bladder cancer: A systematic review and network meta‐analysis
Source: Cancer Med. 2020 Oct 11;9(21):7800–9. doi: 10.1002/cam4.3513 (PMC7643689; doi:10.1002/cam4.3513)

**Quantitative synthesis**

Table S3: Pairwise meta-analysis for all direct comparisons

Table S4: Subgroup and sensitivity analysis on recurrence and progression for direct comparisons

Table S5: Effect sizes estimated using pairwise meta-analysis, indirect meta-analysis, and network meta-analysis on recurrence and progression and consistency test with node-split method

Table S6: Network subgroup analysis on recurrence (reference: transurethral resection of bladder tumor)

Table S7: Network subgroup analysis on progression (reference: transurethral resection of bladder tumor)

Table S8: Network meta-regression for recurrence and progression

Table S9: Ranking analysis for recurrence

Table S10: Ranking analysis for progression

Figure S1: Quality assessment for each study using version 2 of the Cochrane risk-of-bias tool for randomized trials

Figure S2: Quality assessment summary

Figure S3: Cumulative rank for recurrence

Figure S4: Cumulative rank for progression

Table S3: Pairwise meta-analysis for all direct comparisons

| Comparison | Outcome | No. of RCT | HR | 95% CI | *P* | τ^2^ | Q | *I*^2^ | GRADE |
| --- | --- | --- | --- | --- | --- | --- | --- | --- | --- |
| ADM vs THP | Recurrence | 1 | 1.21 | [0.76 to 1.93] | 0.429 | NA | NA | NA | Low |
|  | Progression | 1 | 2.12 | [0.39 to 11.47] | 0.383 | NA | NA | NA | Low |
| ADM vs TURBT | Recurrence | 9 | 0.70 | [0.58 to 0.83] | <0.001 | 0.066 | 52.07 | 77.0% | Very low |
|  | Progression | 4 | 1.05 | [0.71 to 1.55] | 0.806 | 0 | 1.78 | 0.0% | Low |
| BCG vs ADM | Recurrence | 3 | 0.53 | [0.32 to 0.87] | 0.012 | 0.126 | 5.69 | 64.9% | Low |
|  | Progression | 1 | 0.20 | [0.02 to 1.79] | 0.148 | NA | NA | NA | Low |
| BCG vs EPI | Recurrence | 6 | 0.51 | [0.38 to 0.68] | < 0.001 | 0.087 | 15.18 | 60.5% | Low |
|  | Progression | 6 | 0.79 | [0.58 to 1.06] | 0.121 | 0 | 3.80 | 0.0% | Low |
| BCG vs GEM | Recurrence | 3 | 1.04 | [0.38 to 2.89] | 0.944 | 0.695 | 14.57 | 86.3% | Low |
|  | Progression | 2 | 1.01 | [0.47 to 2.16] | 0.981 | 0 | 0.20 | 0.0% | Moderate |
| BCG vs IFN | Recurrence | 1 | 0.57 | [0.38 to 0.85] | 0.007 | NA | NA | NA | Low |
|  | Progression | 1 | 0.69 | [0.24 to 1.95] | 0.485 | NA | NA | NA | Low |
| BCG vs MMC | Recurrence | 9 | 0.95 | [0.76 to 1.19] | 0.672 | 0.110 | 42.49 | 74.1% | Very low |
|  | Progression | 6 | 0.97 | [0.74 to 1.27] | 0.836 | 0.014 | 7.70 | 9.1% | Low |
| BCG vs THP | Recurrence | 1 | 0.31 | [0.14 to 0.68] | 0.003 | NA | NA | NA | Low |
|  | Progression | 1 | 0.42 | [0.04 to 4.66] | 0.479 | NA | NA | NA | Low |
| BCG vs TURBT | Recurrence | 4 | 0.46 | [0.34 to 0.64] | < 0.001 | 0.042 | 5.13 | 41.5% | Moderate |
|  | Progression | 4 | 0.34 | [0.22 to 0.52] | < 0.001 | 0 | 1.26 | 0.0% | Moderate |
| EPI vs ADM | Recurrence | 3 | 0.66 | [0.45 to 0.95] | 0.028 | 0 | 0.95 | 0.0% | Moderate |
|  | Progression | 2 | 0.70 | [0.32 to 1.52] | 0.367 | 0 | 1.70 | 0.0% | Low |
| EPI vs TURBT | Recurrence | 5 | 0.50 | [0.38 to 0.66] | < 0.001 | 0.026 | 6.46 | 22.7% | Moderate |
|  | Progression | 5 | 0.76 | [0.36 to 1.58] | 0.454 | 0.310 | 7.98 | 37.3% | Low |
| IFN vs TURBT | Recurrence | 3 | 0.52 | [0.34 to 0.80] | 0.003 | 0.092 | 6.61 | 39.5% | Low |
|  | Progression | 3 | 0.38 | [0.17 to 0.87] | 0.021 | 0 | 3.25 | 0.0% | Moderate |
| MMC vs ADM | Recurrence | 5 | 0.89 | [0.72 to 1.08] | 0.242 | 0.037 | 13.10 | 38.9% | Low |
|  | Progression | 3 | 0.46 | [0.27 to 0.79] | 0.004 | 0 | 1.56 | 0.0% | Moderate |
| MMC vs EPI | Recurrence | 1 | 0.88 | [0.06 to 13.6] | 0.926 | NA | NA | NA | Low |
|  | Progression | 0 | 1.90 | [1.09 to 3.29] |  |  |  |  |  |
| MMC vs GEM | Recurrence | 1 | 1.51 | [0.90 to 2.51] | 0.022 | NA | NA | NA | Moderate |
|  | Progression | 1 | 0.51 | [0.23 to 1.11] | 0.115 | NA | NA | NA | Low |
| MMC vs IFN | Recurrence | 3 | 1.90 | [0.35 to 10.28] | 0.087 | 0.564 | 19.40 | 79.4% | Low |
|  | Progression | 1 | 1.20 | [0.23 to 6.23] | 0.457 | NA | NA | NA | Low |
| MMC vs THP | Recurrence | 1 | 1.70 | [0.23 to 12.81] | 0.830 | NA | NA | NA | Low |
|  | Progression | 1 | 0.61 | [0.47 to 0.79] | 0.607 | NA | NA | NA | Low |
| MMC vs TURBT | Recurrence | 6 | 0.22 | [0.06 to 0.77] | < 0.001 | 0.053 | 11.13 | 55.1% | Moderate |
|  | Progression | 2 | 0.62 | [0.19 to 2.01] | 0.018 | 0.507 | 2.25 | 55.6% | Low |
| THP vs TURBT | Recurrence | 2 | 1.21 | [0.76 to 1.93] | 0.426 | 0.635 | 8.51 | 88.2% | Very low |
|  | Progression | 0 |  |  |  |  |  |  |  |

Table S4: Subgroup and sensitivity analysis on recurrence and progression for direct comparisons

| Comparison | Outcome | Subgroup | No. of study | HR | 95%CI | τ^2^ | Q | I^2^ |
| --- | --- | --- | --- | --- | --- | --- | --- | --- |
| ADM vs TURBT | Recurrence | Publication year <2000 | 8 | 0.70 | [0.59 to 0.84] | 0.067 | 50.98 | 78.4% |
|  |  | Publication year ≥2000 | 1 | 0.59 | [0.31 to 1.14] | NA | NA | NA |
|  |  | Trial initiation year <1990 | 8 | 0.70 | [0.59 to 0.84] | 0.066 | 49.8 | 77.9% |
|  |  | Trial initiation year ≥1990 | 1 | 0.58 | [0.35 to 0.94] | NA | NA | NA |
|  |  | Conducted in Europe | 2 | 0.86 | [0.72 to 1.03] | 0.004 | 2.40 | 16.6% |
|  |  | Conducted in Africa | 1 | 0.58 | [0.35 to 0.94] | NA | NA | NA |
|  |  | Conducted in Asia | 6 | 0.64 | [0.51 to 0.82] | 0.094 | 47.31 | 83.1% |
|  |  | Single center | 3 | 0.82 | [0.66 to 1.01] | 0.014 | 4.31 | 30.4% |
|  |  | Multiple center | 6 | 0.65 | [0.52 to 0.82] | 0.090 | 47.73 | 83.2% |
|  |  | Sample size <544 | 6 | 0.74 | [0.59 to 0.91] | 0.063 | 34.35 | 79.6% |
|  |  | Sample size ≥544 | 3 | 0.64 | [0.51 to 0.81] | 0.033 | 7.67 | 47.9% |
|  |  | ADM-I | 3 | 0.84 | [0.66 to 1.06] | 0.043 | 11.82 | 74.6% |
|  |  | ADM-M | 6 | 0.63 | [0.50 to 0.79] | 0.076 | 27.60 | 71.0% |
|  |  | AE reported | 5 | 0.58 | [0.41 to 0.82] | 0.136 | 43.97 | 88.6% |
|  |  | AE not reported | 4 | 0.79 | [0.69 to 0.89] | 0.002 | 6.46 | 7.1% |
|  |  | G1/2 | 4 | 0.54 | [0.39 to 0.75] | 0.115 | 18.86 | 73.5% |
|  |  | G1/2/3 | 5 | 0.84 | [0.71 to 0.98] | 0.024 | 16.08 | 62.7% |
|  |  | Quality =7 | 2 | 0.70 | [0.59 to 0.83] | 0 | 0.98 | 0.0% |
|  |  | Quality <7 | 4 | 0.70 | [0.53 to 0.93] | 0.090 | 39.01 | 87.2% |
|  |  | Quality >7 | 3 | 0.63 | [0.48 to 0.82] | 0 | 0.22 | 0.0% |
| ADM vs TURBT | Progression | Publication year <2000 | 3 | 1.00 | [0.66 to 1.52] | 0 | 1.26 | 0.0% |
|  |  | Publication year ≥2000 | 1 | 1.67 | [0.45 to 6.17] | NA | NA | NA |
|  |  | Trial initiation year <1990 | 3 | 1.03 | [0.68 to 1.57] | 0 | 1.70 | 0.0% |
|  |  | Trial initiation year ≥1990 | 1 | 1.22 | [0.38 to 3.90] | NA | NA | NA |
|  |  | Conducted in Europe | 2 | 0.98 | [0.63 to 1.52] | 0 | 1.13 | 0.0% |
|  |  | Conducted in Asia | 1 | 1.22 | [0.38 to 3.9] | NA | NA | NA |
|  |  | Conducted in Africa | 1 | 1.67 | [0.45 to 6.17] | NA | NA | NA |
|  |  | Single center | 3 | 1.20 | [0.75 to 1.92] | 0 | 0.78 | 0.0% |
|  |  | Multiple center | 1 | 0.77 | [0.37 to 1.60] | NA | NA | NA |
|  |  | Sample size <253 | 3 | 1.00 | [0.66 to 1.52] | 0 | 1.26 | 0.0% |
|  |  | Sample size ≥253 | 1 | 1.67 | [0.45 to 6.17] | NA | NA | NA |
|  |  | ADM-I | 1 | 1.34 | [0.64 to 2.80] | NA | NA | NA |
|  |  | ADM-M | 4 | 0.96 | [0.60 to 1.52] | 0 | 1.22 | 0.0% |
|  |  | AE reported | 2 | 0.88 | [0.48 to 1.62] | 0 | 0.44 | 0.0% |
|  |  | AE not reported | 2 | 1.20 | [0.71 to 1.99] | 0 | 0.78 | 0.0% |
|  |  | Quality =7 | 0 |  |  |  |  |  |
|  |  | Quality <7 | 1 | 1.12 | [0.64 to 1.95] | 0 | 0.49 | 0.0% |
|  |  | Quality >7 | 3 | 0.98 | [0.57 to 1.72] | 0 | 1.19 | 0.0% |
| BCG vs ADM | Recurrence | Publication year <2000 | 2 | 0.50 | [0.21 to 1.16] | 0.31 | 4.89 | 79.5% |
|  |  | Publication year ≥2000 | 1 | 0.51 | [0.26 to 0.97] | NA | NA | NA |
|  |  | Trial initiation year <1990 | 2 | 0.50 | [0.21 to 1.16] | 0.308 | 4.89 | 79.5% |
|  |  | Trial initiation year ≥1990 | 1 | 0.51 | [0.26 to 0.97] | NA | NA | NA |
|  |  | Single center | 1 | 0.30 | [0.14 to 0.63] | NA | NA | NA |
|  |  | Multiple center | 2 | 0.70 | [0.60 to 0.81] | 0.000 | 1.00 | 0.3% |
|  |  | Sample size <202 | 2 | 0.50 | [0.21 to 1.16] | 0.308 | 4.89 | 79.5% |
|  |  | Sample size ≥202 | 1 | 0.51 | [0.26 to 0.97] | NA | NA | NA |
|  |  | BCG-M vs ADM-M | 2 | 0.50 | [0.21 to 1.16] | 0.308 | 4.89 | 79.5% |
|  |  | BCG-I vs ADM-M | 1 | 0.51 | [0.26 to 0.97] | NA | NA | NA |
|  |  | G1/2 | 2 | 0.50 | [0.21 to 1.16] | 0.308 | 4.89 | 79.5% |
|  |  | G1/2/3 | 1 | 0.51 | [0.26 to 0.97] | NA | NA | NA |
|  |  | Quality =7 | 2 | 0.70 | [0.6 to 0.81] | 0.000 | 1.00 | 0.3% |
|  |  | Quality <7 | 1 | 0.30 | [0.14 to 0.63] | NA | NA | NA |
|  |  | Quality >7 | 0 |  |  |  |  |  |
| BCG vs EPI | Recurrence | Publication year <2000 | 2 | 0.78 | [0.53 to 1.15] | 0 | 0.07 | 0.0% |
|  |  | Publication year ≥2000 | 4 | 0.43 | [0.30 to 0.62] | 0.105 | 11.74 | 65.9% |
|  |  | Trial initiation year <2000 | 5 | 0.58 | [0.46 to 0.73] | 0.024 | 6.07 | 34.1% |
|  |  | Trial initiation year ≥2000 | 1 | 0.29 | [0.11 to 0.76] | 0.355 | 3.44 | 70.9% |
|  |  | Conducted in Europe | 4 | 0.61 | [0.46 to 0.80] | 0.030 | 4.73 | 36.6% |
|  |  | Conducted in Asia | 2 | 0.36 | [0.20 to 0.64] | 0.157 | 5.14 | 61.1% |
|  |  | Single center | 2 | 0.61 | [0.35 to 1.04] | 0.093 | 2.59 | 61.3% |
|  |  | Multiple center | 4 | 0.45 | [0.30 to 0.69] | 0.142 | 12.50 | 68.0% |
|  |  | Sample size <957 | 5 | 0.47 | [0.32 to 0.69] | 0.139 | 13.17 | 62.0% |
|  |  | Sample size ≥957 | 1 | 0.62 | [0.50 to 0.77] | NA | NA | NA |
|  |  | BCG (Pasteur) | 1 | 0.73 | [0.41 to 1.32] | NA | NA | NA |
|  |  | BCG (Tice) | 2 | 0.64 | [0.53 to 0.79] | 0 | 0.92 | 0.0% |
|  |  | BCG (Connaught) | 3 | 0.37 | [0.25 to 0.55] | 0.067 | 5.21 | 42.4% |
|  |  | AE reported | 4 | 0.46 | [0.28 to 0.77] | 0.228 | 13.11 | 69.5% |
|  |  | AE not reported | 2 | 0.57 | [0.45 to 0.73] | 0.008 | 1.30 | 22.9% |
|  |  | Quality =7 | 2 | 0.56 | [0.37 to 0.85] | 0.030 | 1.44 | 30.7% |
|  |  | Quality <7 | 0 |  |  |  |  |  |
|  |  | Quality >7 | 4 | 0.47 | [0.30 to 0.71] | 0.154 | 13.72 | 70.9% |
| BCG vs EPI | Progression | Publication year <2000 | 2 | 1.09 | [0.53 to 2.27] | 0 | 0.67 | 0.0% |
|  |  | Publication year ≥2000 | 4 | 0.73 | [0.53 to 1.02] | 0 | 2.20 | 0.0% |
|  |  | Trial initiation year <2000 | 5 | 0.83 | [0.61 to 1.13] | 0 | 1.91 | 0.0% |
|  |  | Trial initiation year ≥2000 | 1 | 0.33 | [0.09 to 1.19] | NA | NA | NA |
|  |  | Single center | 2 | 1.02 | [0.59 to 1.75] | 0 | 0.63 | 0.0% |
|  |  | Multiple center | 4 | 0.70 | [0.49 to 1.01] | 0 | 1.94 | 0.0% |
|  |  | Conducted in Asia | 2 | 0.63 | [0.25 to 1.52] | 0.191 | 1.69 | 40.7% |
|  |  | Conducted in Europe | 4 | 0.82 | [0.58 to 1.16] | 0 | 1.90 | 0.0% |
|  |  | Sample size <957 | 5 | 0.76 | [0.52 to 1.11] | 0 | 3.69 | 0.0% |
|  |  | Sample size ≥957 | 1 | 0.84 | [0.51 to 1.40] | NA | NA | NA |
|  |  | BCG (Pasteur) | 1 | 0.72 | [0.21 to 2.46] | NA | NA | NA |
|  |  | BCG (Tice) | 2 | 0.95 | [0.61 to 1.48] | 0 | 0.83 | 0.0% |
|  |  | BCG (Connaught) | 3 | 0.66 | [0.43 to 1.03] | 0 | 1.72 | 0.0% |
|  |  | AE reported | 4 | 0.72 | [0.44 to 1.19] | 0.040 | 3.51 | 14.5% |
|  |  | AE not reported | 2 | 0.85 | [0.57 to 1.28] | 0 | 0.00 | 0.0% |
|  |  | Quality =7 | 2 | 0.83 | [0.45 to 1.49] | 0 | 0.06 | 0.0% |
|  |  | Quality <7 | 0 |  |  |  |  |  |
|  |  | Quality >7 | 4 | 0.77 | [0.51 to 1.15] | 0.034 | 3.70 | 19.0% |
| BCG vs GEM | Recurrence | Single center | 1 | 0.45 | [0.21 to 0.96] | NA | NA | NA |
|  |  | Multiple center | 2 | 1.55 | [0.61 to 4.01] | 0.371 | 4.72 | 78.8% |
|  |  | BCG (Tice) | 1 | 0.45 | [0.21 to 0.96] | NA | NA | NA |
|  |  | BCG (Connaught) | 2 | 1.55 | [0.61 to 4.01] | 0.371 | 4.72 | 78.8% |
|  |  | High risk | 2 | 1.07 | [0.21 to 0.18] | 1.321 | 13.31 | 92.5% |
|  |  | Intermediate risk | 1 | 0.92 | [0.44 to 1.92] | NA | NA | NA |
|  |  | Quality =7 | 1 | 0.45 | [0.21 to 0.96] | NA | NA | NA |
|  |  | Quality <7 | 1 | 0.92 | [0.44 to 1.92] | NA | NA | NA |
|  |  | Quality >7 | 1 | 2.44 | [1.49 to 3.97] | NA | NA | NA |
| BCG vs MMC | Recurrence | Publication year <2000 | 5 | 0.99 | [0.76 to 1.30] | 0.079 | 18.43 | 72.9% |
|  |  | Publication year ≥2000 | 4 | 0.91 | [0.61 to 1.38] | 0.202 | 23.37 | 78.6% |
|  |  | Trial initiation year <1990 | 6 | 0.91 | [0.69 to 1.20] | 0.103 | 25.40 | 76.4% |
|  |  | Trial initiation year ≥1990 | 3 | 1.03 | [0.68 to 1.57] | 0.174 | 16.92 | 76.4% |
|  |  | Conducted in Europe | 7 | 0.99 | [0.76 to 1.27] | 0.128 | 37.8 | 76.2% |
|  |  | Conducted in Oceania | 1 | 0.70 | [0.52 to 0.93] | NA | NA | NA |
|  |  | Conducted in North America | 1 | 0.96 | [0.61 to 1.51] | NA | NA | NA |
|  |  | Single center | 1 | 0.84 | [0.48 to 1.49] | NA | NA | NA |
|  |  | Multiple center | 8 | 0.96 | [0.76 to 1.22] | 0.118 | 42.34 | 76.4% |
|  |  | Sample size <261 | 2 | 0.63 | [0.37 to 1.07] | 0.073 | 1.99 | 49.7% |
|  |  | Sample size ≥261 | 7 | 1.02 | [0.81 to 1.28] | 0.101 | 35.10 | 74.4% |
|  |  | BCG (RIVM) | 3 | 1.31 | [0.96 to 1.79] | 0.057 | 7.07 | 57.5% |
|  |  | BCG (TICE) | 3 | 0.98 | [0.56 to 1.72] | 0.203 | 13.04 | 84.7% |
|  |  | BCG (Connaught) | 1 | 0.96 | [0.61 to 1.51] | NA | NA | NA |
|  |  | BCG (Danish) | 1 | 0.71 | [0.52 to 0.97] | NA | NA | NA |
|  |  | BCG (Pasteur) | 1 | 0.49 | [0.3 to 0.80] | NA | NA | NA |
|  |  | BCG-I vs MMC-I | 2 | 1.62 | [0.80 to 3.25] | 0.213 | 5.56 | 82.0% |
|  |  | BCG-I vs MMC-M | 3 | 1.21 | [0.97 to 1.51] | 0.010 | 3.69 | 18.8% |
|  |  | BCG-M vs MMC-M | 5 | 0.70 | [0.61 to 0.82] | 0.000 | 4.01 | 0.0% |
|  |  | AE reported | 8 | 1.04 | [0.81 to 1.25] | 0.094 | 35.36 | 71.7% |
|  |  | AE not reported | 1 | 0.49 | [0.30 to 0.80] | NA | NA | NA |
|  |  | G1/2 | 2 | 0.77 | [0.61 to 0.99] | 0.000 | 1.37 | 0.0% |
|  |  | G1/2/3 | 4 | 0.90 | [0.56 to 1.43] | 0.194 | 20.65 | 85.5% |
|  |  | Quality =7 | 0 |  |  |  |  |  |
|  |  | Quality <7 | 3 | 1.03 | [0.69 to 1.55] | 0.135 | 14.82 | 79.8% |
|  |  | Quality >7 | 6 | 0.90 | [0.70 to 1.17] | 0.089 | 21.99 | 68.2% |
| BCG vs MMC | Progression | Publication year <2000 | 3 | 1.11 | [0.75 to 1.63] | 0.005 | 3.08 | 2.7% |
|  |  | Publication year ≥2000 | 3 | 0.87 | [0.58 to 1.30] | 0.034 | 3.78 | 20.6% |
|  |  | Trial initiation year <1990 | 5 | 0.90 | [0.64 to 1.30] | 0.050 | 6.70 | 25.4% |
|  |  | Trial initiation year ≥1990 | 1 | 1.17 | [0.71 to 1.93] | NA | NA | NA |
|  |  | Conducted in North America | 1 | 0.79 | [0.42 to 1.49] | NA | NA | NA |
|  |  | Conducted in Europe | 5 | 1.01 | [0.74 to 1.38] | 0.031 | 7.23 | 17.0% |
|  |  | Sample size <344 | 3 | 0.88 | [0.41 to 1.88] | 0.305 | 6.42 | 68.8% |
|  |  | Sample size ≥344 | 3 | 1.01 | [0.72 to 1.42] | 0 | 1.15 | 0.0% |
|  |  | BCG-I vs MMC-I | 1 | 1.80 | [0.90 to 3.60] | NA | NA | NA |
|  |  | BCG-I vs MMC-M | 1 | 1.01 | [0.52 to 1.99] | 0 | 0.00 | 0.0% |
|  |  | BCG-M vs MMC-M | 4 | 0.85 | [0.63 to 1.15] | 0 | 3.84 | 0.0% |
|  |  | BCG (TICE) | 2 | 0.85 | [0.50 to 1.45] | 0 | 0.18 | 0.0% |
|  |  | BCG (RIVM) | 2 | 1.48 | [0.84 to 2.56] | 0 | 0.95 | 0.0% |
|  |  | BCG (Danish) | 1 | 0.74 | [0.44 to 1.26] | NA | NA | NA |
|  |  | BCG (Pasteur) | 1 | 0.41 | [0.14 to 1.23] | NA | NA | NA |
|  |  | AE reported | 4 | 1.13 | [0.84 to 1.54] | 0 | 3.36 | 0.0% |
|  |  | AE not reported | 2 | 0.66 | [0.41 to 1.07] | 0 | 0.90 | 0.0% |
|  |  | Quality =7 | 0 |  |  |  |  |  |
|  |  | Quality <7 | 3 | 1.03 | [0.57 to 1.86] | 0.153 | 5.15 | 41.7% |
|  |  | Quality >7 | 3 | 0.90 | [0.66 to 1.23] | 0 | 2.00 | 0.0% |
| BCG vs TURBT | Recurrence | Single center | 2 | 0.43 | [0.26 to 0.73] | 0.094 | 3.02 | 66.9% |
|  |  | Multiple center | 2 | 0.52 | [0.33 to 0.81] | 0.024 | 1.28 | 21.6% |
|  |  | Sample size <337 | 3 | 0.42 | [0.30 to 0.59] | 0.033 | 3.08 | 35.1% |
|  |  | Sample size ≥337 | 1 | 0.62 | [0.39 to 0.99] | NA | NA | NA |
|  |  | Conducted in Europe | 3 | 0.42 | [0.30 to 0.59] | 0.033 | 3.08 | 35.1% |
|  |  | Conducted in Oceania | 1 | 0.62 | [0.39 to 0.99] | NA | NA | NA |
|  |  | BCG (Pasteur) | 3 | 0.42 | [0.30 to 0.59] | 0.033 | 3.08 | 35.1% |
|  |  | BCG (Connaught) | 1 | 0.62 | [0.39 to 0.90] | NA | NA | NA |
|  |  | BCG-I | 2 | 0.59 | [0.43 to 0.82] | 0 | 0.06 | 0.0% |
|  |  | BCG-M | 2 | 0.35 | [0.25 to 0.49] | 0 | 0.13 | 0.0% |
|  |  | Quality =7 | 1 | 0.39 | [0.20 to 0.76] | NA | NA | NA |
|  |  | Quality <7 | 0 |  |  |  |  |  |
|  |  | Quality >7 | 3 | 0.48 | [0.33 to 0.71] | 0.070 | 4.84 | 58.7% |
| BCG vs TURBT | Progression | Conducted in Europe | 3 | 0.27 | [0.15 to 0.49] | 0 | 0.04 | 0.0% |
|  |  | Conducted in North America | 1 | 0.43 | [0.23 to 0.79] | NA | NA | NA |
|  |  | Single center | 3 | 0.35 | [0.22 to 0.54] | 0 | 1.20 | 0.0% |
|  |  | Multiple center | 1 | 0.30 | [0.09 to 0.96] | NA | NA | NA |
|  |  | BCG-I | 2 | 0.36 | [0.22 to 0.59] | 0 | 0.88 | 0.0% |
|  |  | BCG-M | 2 | 0.27 | [0.12 to 0.64] | 0 | 0.04 | 0.0% |
|  |  | AE reported | 3 | 0.27 | [0.15 to 0.49] | 0 | 0.04 | 0.0% |
|  |  | AE not reported | 1 | 0.43 | [0.23 to 0.79] | NA | NA | NA |
|  |  | Quality =7 | 2 | 0.40 | [0.23 to 0.68] | 0 | 0.32 | 0.0% |
|  |  | Quality <7 | 0 |  |  |  |  |  |
|  |  | Quality >7 | 1 | 0.26 | [0.13 to 0.52] | NA | NA | NA |
| EPI vs ADM | Recurrence | Conducted in Asia | 2 | 0.76 | [0.41 to 1.43] | 0 | 0.42 | 0.0% |
|  |  | Conducted in Africa | 1 | 0.60 | [0.38 to 0.96] | NA | NA | NA |
|  |  | Single center | 1 | 0.60 | [0.38 to 0.96] | NA | NA | NA |
|  |  | Multiple center | 2 | 0.76 | [0.41 to 1.43] | 0 | 0.42 | 0.0% |
|  |  | Sample size <150 | 1 | 0.91 | [0.40 to 2.08] | NA | NA | NA |
|  |  | Sample size ≥150 | 2 | 0.60 | [0.39 to 0.91] | 0 | 0.16 | 0.0% |
|  |  | G1/2 | 1 | 0.91 | [0.40 to 2.08] | NA | NA | NA |
|  |  | G1/2/3 | 2 | 0.60 | [0.39 to 0.91] | 0 | 0.16 | 0.0% |
|  |  | Quality =7 | 2 | 0.76 | [0.41 to 1.43] | 0 | 0.42 | 0.0% |
|  |  | Quality <7 | 0 |  |  |  |  |  |
|  |  | Quality >7 | 1 | 0.60 | [0.38 to 0.96] | NA | NA | NA |
| EPI vs TURBT | Recurrence | Conducted in Europe | 2 | 0.53 | [0.33 to 0.87] | 0 | 0.00 | 0.0% |
|  |  | Conducted in Asia | 1 | 0.89 | [0.50 to 1.57] | NA | NA | NA |
|  |  | Conducted in Africa | 2 | 0.41 | [0.30 to 0.55] | 0 | 0.87 | 0.0% |
|  |  | Single center | 4 | 0.50 | [0.36 to 0.68] | 0.051 | 6.40 | 37.5% |
|  |  | Multiple center | 1 | 0.54 | [0.29 to 1.01] | NA | NA | NA |
|  |  | Sample size <181 | 2 | 0.73 | [0.45 to 1.20] | 0.013 | 1.11 | 9.7% |
|  |  | Sample size ≥181 | 3 | 0.43 | [0.33 to 0.57] | 0 | 1.45 | 0.0% |
|  |  | G2/3 | 1 | 0.49 | [0.30 to 0.82] | NA | NA | NA |
|  |  | G1/2/3 | 4 | 0.50 | [0.36 to 0.71] | 0.058 | 6.46 | 38.1% |
|  |  | AE reported | 3 | 0.42 | [0.31 to 0.58] | 0 | 1.44 | 0.0% |
|  |  | AE not reported | 2 | 0.65 | [0.36 to 1.16] | 0.098 | 2.29 | 56.4% |
|  |  | Quality =7 | 2 | 0.70 | [0.43 to 1.15] | 0.031 | 1.34 | 25.4% |
|  |  | Quality <7 | 1 | 0.49 | [0.30 to 0.82] | NA | NA | NA |
|  |  | Quality >7 | 2 | 0.39 | [0.28 to 0.56] | 0 | 0.74 | 0.0% |
| EPI vs TURBT | Progression | Conducted in Europe | 2 | 0.43 | [0.18 to 1.06] | 0 | 0.04 | 0.0% |
|  |  | Conducted in Asia | 1 | 6.69 | [0.89 to 50.4] | NA | NA | NA |
|  |  | Conducted in Africa | 2 | 0.76 | [0.35 to 1.63] | 0 | 1.99 | 0.0% |
|  |  | Single center | 4 | 0.92 | [0.38 to 2.20] | 0.358 | 6.30 | 36.5% |
|  |  | Multiple center | 1 | 0.41 | [0.15 to 1.14] | NA | NA | NA |
|  |  | Sample size <181 | 2 | 1.82 | [0.15 to 22.42] | 2.292 | 3.27 | 69.4% |
|  |  | Sample size ≥181 | 3 | 0.61 | [0.33 to 1.13] | 0 | 2.88 | 0.0% |
|  |  | AE reported | 3 | 0.64 | [0.34 to 1.21] | 0 | 2.51 | 0.0% |
|  |  | AE not reported | 2 | 1.46 | [0.09 to 25.03] | 3.338 | 4.85 | 79.4% |
|  |  | Quality =7 | 2 | 1.43 | [0.09 to 21.76] | 3.230 | 5.85 | 82.9% |
|  |  | Quality <7 | 1 | 0.37 | [0.07 to 1.84] | NA | NA | NA |
|  |  | Quality >7 | 2 | 0.84 | [0.38 to 1.88] | 0 | 1.30 | 0.0% |
| IFN vs TURBT | Recurrence | Publication year <2000 | 2 | 0.57 | [0.36 to 0.90] | 0.094 | 5.27 | 43.0% |
|  |  | Publication year ≥2000 | 1 | 0.31 | [0.11 to 0.86] | NA | NA | NA |
|  |  | Single center | 2 | 0.57 | [0.36 to 0.90] | 0.094 | 5.27 | 43.0% |
|  |  | Multiple center | 1 | 0.31 | [0.11 to 0.86] | NA | NA | NA |
|  |  | IFN-I | 1 | 0.31 | [0.11 to 0.86] | NA | NA | NA |
|  |  | IFN-M | 2 | 0.57 | [0.36 to 0.90] | 0.094 | 5.27 | 43.0% |
|  |  | G2 | 1 | 1.00 | [0.54 to 1.84] | NA | NA | NA |
|  |  | G2/3 | 2 | 0.43 | [0.30 to 0.64] | 0 | 1.40 | 0.0% |
|  |  | Quality =7 | 0 |  |  |  |  |  |
|  |  | Quality <7 | 2 | 0.60 | [0.19 to 1.88] | 0.501 | 3.74 | 73.2% |
|  |  | Quality >7 | 1 | 0.46 | [0.30 to 0.69] | 0 | 0.92 | 0.0% |
| IFN vs TURBT | Progression | Publication year <2000 | 2 | 0.34 | [0.15 to 0.82] | 0 | 2.66 | 0.0% |
|  |  | Publication year ≥2000 | 1 | 1.07 | [0.07 to 16.95] | NA | NA | NA |
|  |  | Single center | 2 | 0.34 | [0.15 to 0.82] | 0 | 2.66 | 0.0% |
|  |  | Multiple center | 1 | 1.07 | [0.07 to 16.95] | NA | NA | NA |
|  |  | IFN-I | 1 | 1.07 | [0.07 to 16.95] | NA | NA | NA |
|  |  | IFN-M | 2 | 0.34 | [0.15 to 0.82] | 0 | 2.66 | 0.0% |
|  |  | G2 | 1 | 0.99 | [0.13 to 7.61] | NA | NA | NA |
|  |  | G2/3 | 2 | 0.32 | [0.13 to 0.78] | 0 | 2.24 | 0.0% |
|  |  | Quality =7 | 0 |  |  |  |  |  |
|  |  | Quality <7 | 2 | 1.02 | [0.20 to 5.26] | 0 | 0.00 | 0.0% |
|  |  | Quality >7 | 1 | 0.27 | [0.11 to 0.71] | NA | NA | NA |
| MMC vs ADM | Recurrence | Publication year <1990 | 4 | 0.97 | [0.76 to 1.23] | 0.041 | 9.25 | 45.9% |
|  |  | Publication year ≥1990 | 1 | 0.68 | [0.49 to 0.97] | NA | NA | NA |
|  |  | Conducted in Europe | 3 | 1.02 | [0.85 to 1.21] | 0 | 3.04 | 0.0% |
|  |  | Conducted in Asia | 2 | 0.62 | [0.44 to 0.86] | 0.007 | 3.17 | 5.5% |
|  |  | Sample size <597 | 2 | 0.76 | [0.13 to 4.35] | 1.411 | 8.38 | 88.1% |
|  |  | Sample size ≥597 | 3 | 0.91 | [0.78 to 1.07] | 0 | 4.72 | 0.0% |
|  |  | MMC-I vs ADM-I | 1 | 1.02 | [0.80 to 1.30] | NA | NA | NA |
|  |  | MMC-M vs ADM-M | 4 | 0.81 | [0.61 to 1.08] | 0.065 | 11.13 | 46.1% |
|  |  | G1/2 | 3 | 1.02 | [0.85 to 1.21] | 0 | 3.04 | 0.0% |
|  |  | G1/2/3 | 2 | 0.62 | [0.44 to 0.86] | 0.007 | 3.17 | 5.5% |
|  |  | AE reported | 2 | 0.76 | [0.13 to 4.35] | 1.411 | 8.38 | 88.1% |
|  |  | AE not reported | 3 | 0.91 | [0.78 to 1.07] | 0 | 4.72 | 0.0% |
|  |  | Quality =7 | 3 | 0.92 | [0.73 to 1.16] | 0.028 | 6.64 | 39.8% |
|  |  | Quality <7 | 2 | 0.83 | [0.53 to 1.28] | 0.096 | 5.72 | 47.5% |
|  |  | Quality >7 | 0 |  |  |  |  |  |
| MMC vs IFN | Recurrence | Publication year <2000 | 1 | 0.68 | [0.48 to 0.97] | NA | NA | NA |
|  |  | Publication year ≥2000 | 2 | 0.42 | [0.12 to 1.54] | 1.436 | 19.30 | 84.5% |
|  |  | Conducted in North America | 1 | 0.68 | [0.48 to 0.97] | NA | NA | NA |
|  |  | Conducted in Europe | 2 | 0.42 | [0.12 to 1.54] | 1.436 | 19.30 | 84.5% |
|  |  | Single center | 1 | 0.23 | [0.12 to 0.47] | NA | NA | NA |
|  |  | Multiple center | 2 | 1.08 | [0.41 to 2.83] | 0.416 | 0.6449 | 86.6% |
|  |  | Sample size <287 | 1 | 0.68 | [0.48 to 0.97] | NA | NA | NA |
|  |  | Sample size ≥287 | 2 | 0.42 | [0.12 to 1.54] | 1.436 | 19.30 | 84.5% |
|  |  | MMC-I vs IFN-I | 2 | 0.36 | [0.17 to 0.76] | 0.332 | 7.58 | 60.4% |
|  |  | MMC-M vs IFN-M | 1 | 1.82 | [0.99 to 3.35] | NA | NA | NA |
|  |  | Quality =7 | 0 |  |  |  |  |  |
|  |  | Quality <7 | 0 |  |  |  |  |  |
|  |  | Quality >7 | 3 | 0.51 | [0.23 to 1.11] | 0.564 | 19.40 | 79.4% |
| MMC vs TURBT | Recurrence | Publication year <1990 | 4 | 0.68 | [0.48 to 0.96] | 0.075 | 7.67 | 60.9% |
|  |  | Publication year ≥1990 | 2 | 0.50 | [0.39 to 0.66] | 0 | 0.00 | 0.0% |
|  |  | Conducted in Europe | 1 | 0.50 | [0.36 to 0.70] | NA | NA | NA |
|  |  | Conducted in Asia | 4 | 0.68 | [0.48 to 0.96] | 0.075 | 7.67 | 60.9% |
|  |  | Conducted in Oceania | 1 | 0.51 | [0.32 to 0.79] | NA | NA | NA |
|  |  | Single center | 2 | 0.71 | [0.34 to 1.48] | 0.233 | 5.83 | 82.8% |
|  |  | Multiple center | 4 | 0.58 | [0.44 to 0.77] | 0.034 | 5.27 | 43.1% |
|  |  | MMC-I | 4 | 0.64 | [0.47 to 0.87] | 0.055 | 7.08 | 57.6% |
|  |  | MMC-M | 2 | 0.51 | [0.25 to 1.04] | 0.199 | 3.95 | 74.7% |
|  |  | AE reported | 3 | 0.58 | [0.31 to 1.08] | 0.233 | 8.52 | 76.5% |
|  |  | AE not reported | 3 | 0.63 | [0.50 to 0.78] | 0.008 | 2.54 | 21.2% |
|  |  | Quality =7 | 2 | 0.70 | [0.55 to 0.90] | 0 | 0.00 | 0.0% |
|  |  | Quality <7 | 2 | 0.45 | [0.32 to 0.63] | 0.010 | 1.16 | 13.7% |
|  |  | Quality >7 | 2 | 0.73 | [0.35 to 1.51] | 0.214 | 4.54 | 78.0% |

Table S5: Effect sizes estimated using pairwise meta-analysis, indirect meta-analysis, and network meta-analysis on recurrence and progression and consistency test with node-split method

| Outcome | Comparison | Direct effect | Indirect effect | Mix effect | *P*-value |
| --- | --- | --- | --- | --- | --- |
| Recurrence | BCG vs ADM | 0.48[0.27 to 0.82] | 0.64[0.48 to 0.85] | 0.61[0.47 to 0.78] | 0.340 |
|  | EPI vs ADM | 0.68[0.41 to 1.15] | 1.14[0.79 to 1.65] | 0.95[0.70 to 1.28] | 0.114 |
|  | MMC vs ADM | 0.90[0.67 to 1.21] | 0.67[0.48 to 0.95] | 0.79[0.63 to 0.99] | 0.195 |
|  | THP vs ADM | 0.83[0.33 to 2.03] | 0.94[0.49 to 1.82] | 0.99[0.59 to 1.65] | 0.807 |
|  | TURBT vs ADM | 1.54[1.22 to 1.95] | 1.09[0.71 to 1.65] | 1.43[1.17 to 1.75] | 0.155 |
|  | EPI vs BCG | 1.97[1.42 to 2.72] | 1.05[0.68 to 1.63] | 1.55[1.19 to 2.05] | 0.025 |
|  | GEM vs BCG | 0.90[0.50 to 1.62] | 0.67[0.25 to 1.79] | 0.83[0.51 to 1.38] | 0.618 |
|  | IFN vs BCG | 1.77[0.73 to 4.48] | 0.93[0.62 to 1.39] | 1.04[0.72 to 1.49] | 0.189 |
|  | MMC vs BCG | 1.03[0.81 to 1.30] | 1.99[1.42 to 2.72] | 1.28[1.05 to 1.58] | 0.002 |
|  | THP vs BCG | 3.32[1.06 to 9.97] | 1.49[0.76 to 3.00] | 1.62[0.95 to 2.72] | 0.233 |
|  | TURBT vs BCG | 2.16[1.35 to 3.32] | 2.41[1.82 to 3.32] | 2.34[1.86 to 3.00] | 0.672 |
|  | MMC vs EPI | 0.85[0.05 to 13.46] | 0.83[0.61 to 1.13] | 0.83[0.61 to 1.13] | 0.981 |
|  | TURBT vs EPI | 1.99[1.34 to 3.00] | 1.11[0.73 to 1.68] | 1.51[1.14 to 1.99] | 0.046 |
|  | MMC vs GEM | 1.92[0.73 to 4.95] | 1.42[0.76 to 0.38] | 0.64[0.92 to 0.38] | 0.619 |
|  | MMC vs IFN | 1.75[1.06 to 3.00] | 0.92[0.58 to 1.46] | 1.23[0.88 to 1.77] | 0.066 |
|  | TURBT vs IFN | 1.93[1.20 to 3.00] | 2.66[1.63 to 4.48] | 2.25[1.62 to 3.32] | 0.367 |
|  | THP vs MMC | 0.85[0.14 to 4.95] | 1.31[0.75 to 2.27] | 1.26[0.74 to 2.14] | 0.655 |
|  | TURBT vs MMC | 1.57[1.17 to 2.10] | 2.23[1.65 to 3.00] | 1.82[1.46 to 2.27] | 0.099 |
|  | TURBT vs THP | 1.48[0.76 to 3.00] | 1.42[0.65 to 3.00] | 1.45[0.88 to 2.39] | 0.945 |
| Progression | BCG vs ADM | 0.20[0.05 to 0.80] | 0.38[0.25 to 0.56] | 0.37[0.25 to 0.54] | 0.386 |
|  | EPI vs ADM | 0.76[0.43 to 1.28] | 0.50[0.30 to 0.83] | 0.54[0.37 to 0.84] | 0.251 |
|  | MMC vs ADM | 0.44[0.25 to 0.79] | 0.33[0.20 to 0.55] | 0.38[0.25 to 0.55] | 0.468 |
|  | THP vs ADM | 0.45[0.07 to 2.53] | 0.22[0.02 to 1.86] | 0.37[0.10 to 1.42] | 0.643 |
|  | TURBT vs ADM | 0.94[0.63 to 1.39] | 1.34[0.67 to 2.61] | 1.06[0.75 to 1.49] | 0.394 |
|  | EPI vs BCG | 1.28[0.92 to 1.80] | 2.69[1.32 to 4.95] | 1.45[1.07 to 1.99] | 0.061 |
|  | GEM vs BCG | 1.00[0.44 to 2.29] | 0.66[0.33 to 1.32] | 0.78[0.47 to 1.32] | 0.442 |
|  | IFN vs BCG | 1.39[0.46 to 4.48] | 0.95[0.40 to 2.48] | 1.08[0.58 to 2.10] | 0.571 |
|  | MMC vs BCG | 1.03[0.76 to 1.38] | 0.99[0.59 to 1.68] | 1.02[0.80 to 1.32] | 0.903 |
|  | THP vs BCG | 2.48[0.25 to 24.53] | 0.59[0.07 to 4.48] | 0.99[0.27 to 4.06] | 0.361 |
|  | TURBT vs BCG | 3.00[1.88 to 4.95] | 2.69[1.70 to 4.48] | 3.00[2.08 to 4.06] | 0.755 |
|  | TURBT vs EPI | 1.39[0.76 to 2.46] | 2.64[1.54 to 4.48] | 1.97[1.35 to 2.72] | 0.117 |
|  | MMC vs GEM | 1.52[0.79 to 3.00] | 1.02[0.44 to 2.34] | 0.76[0.79 to 2.16] | 0.433 |
|  | MMC vs IFN | 1.84[0.37 to 9.97] | 0.83[0.41 to 1.68] | 0.94[0.47 to 1.77] | 0.403 |
|  | TURBT vs IFN | 2.59[1.09 to 6.05] | 2.56[0.97 to 6.69] | 2.61[1.38 to 4.95] | 0.967 |
|  | THP vs MMC | 0.52[0.07 to 4.48] | 1.55[0.3 to 11.02] | 0.97[0.27 to 3.67] | 0.456 |
|  | TURBT vs MMC | 5.47[2.72 to 11.02] | 2.29[1.60 to 3.32] | 2.72[1.97 to 4.06] | 0.025 |

Table S6: Network subgroup analysis on recurrence (reference: transurethral resection of bladder tumor)

| Criteria | Subgroup | ADM | BCG | EPI | GEM | IFN | MMC | THP |
| --- | --- | --- | --- | --- | --- | --- | --- | --- |
| Year | <1995 | 0.71[0.56 to 0.90] | 0.39[0.27 to 0.58] | 0.54[0.3 to 0.90] | NA | 1.02[0.41 to 2.46] | 0.70[0.50 to 0.95] | 0.70[0.42 to 1.15] |
|  | ≥1995 | 0.71[0.49 to 1.05] | 0.40[0.27 to 0.58] | 0.68[0.47 to 1.01] | 0.33[0.18 to 0.61] | 0.38[0.25 to 0.56] | 0.49[0.33 to 0.70] | NA |
| Sample size | <253 | 0.67[0.41 to 1.13] | 0.37[0.25 to 0.52] | 0.67[0.44 to 1.03] | 0.33[0.17 to 0.65] | 0.39[0.25 to 0.61] | 0.63[0.41 to 0.98] | 0.53[0.25 to 1.13] |
|  | ≥253 | 0.73[0.61 to 0.87] | 0.55[0.42 to 0.72] | 0.57[0.38 to 0.85] | NA | 0.85[0.43 to 1.68] | 0.58[0.47 to 0.72] | 1.08[0.57 to 2.05] |
| Center | Single | 0.84[0.61 to 1.21] | 0.38[0.25 to 0.56] | 0.55[0.38 to 0.79] | 0.84[0.27 to 2.61] | 0.41[0.25 to 0.63] | 0.70[0.47 to 1.07] | 0.59[0.30 to 1.11] |
|  | Multiple | 0.64[0.50 to 0.83] | 0.46[0.33 to 0.62] | 0.84[0.54 to 1.30] | 0.27[0.15 to 0.52] | 0.55[0.33 to 0.92] | 0.53[0.39 to 0.70] | 1.08[0.47 to 2.48] |
| AE | No reported | 0.76[0.66 to 0.89] | 0.33[0.22 to 0.51] | 0.61[0.43 to 0.89] | NA | 0.61[0.33 to 1.15] | 0.67[0.55 to 0.79] | 1.08[0.69 to 1.68] |
|  | Reported | 0.68[0.49 to 0.94] | 0.41[0.30 to 0.58] | 0.63[0.41 to 0.95] | 0.33[0.18 to 0.67] | 0.37[0.25 to 0.57] | 0.49[0.33 to 0.70] | 0.67[0.37 to 1.21] |
| RoB2 | ≤7 | 0.70[0.55 to 0.88] | 0.44[0.30 to 0.61] | 0.65[0.43 to 0.98] | 0.68[0.30 to 1.51] | 0.70[0.38 to 1.27] | 0.54[0.41 to 0.71] | 0.70[0.42 to 1.14] |
|  | >7 | 0.69[0.43 to 1.13] | 0.44[0.30 to 0.64] | 0.69[0.45 to 1.07] | 0.22[0.10 to 0.50] | 0.37[0.20 to 0.56] | 0.53[0.37 to 0.80] | NA |
| Grade | G12 | 0.55[0.38 to 0.78] | 0.43[0.25 to 0.78] | 0.52[0.17 to 1.67] | 0.67[0.20 to 2.14] | 0.39[0.25 to 0.61] | 0.62[0.43 to 0.89] | 0.33[0.11 to 0.99] |
|  | G123 | 0.83[0.67 to 1.03] | 0.46[0.37 to 0.59] | 0.58[0.43 to 0.77] | 0.30[0.18 to 0.54] | 0.74[0.44 to 1.22] | 0.53[0.40 to 0.69] | 0.96[0.58 to 1.62] |
| Schedule | Induction | 0.83[0.54 to 1.31] | 0.70[0.42 to 1.15] | NA | NA | 0.33[0.17 to 0.67] | 0.64[0.42 to 0.97] | 1.08[0.40 to 3.00] |
|  | Maintenance | 0.63[0.51 to 0.76] | 0.33[0.25 to 0.42] | 0.53[0.41 to 0.69] | 0.27[0.18 to 0.46] | 0.51[0.37 to 0.73] | 0.52[0.40 to 0.66] | 0.50[0.27 to 0.85] |

Table S7: Network subgroup analysis on progression (reference: transurethral resection of bladder tumor)

| Criteria | Subgroup | ADM | BCG | EPI | GEM | IFN | MMC | THP |
| --- | --- | --- | --- | --- | --- | --- | --- | --- |
| Year | <2000 | 0.88[0.61 to 1.30] | 0.37[0.25 to 0.52] | 0.54[0.33 to 0.90] | NA | 0.40[0.20 to 0.83] | 0.30[0.20 to 0.47] | 0.33[0.09 to 1.26] |
|  | ≥2000 | 1.65[2.51 to 6.69] | 1.57[16.44 to 49.4] | 2.18[12.18 to 73.7] | 1.35[20.09 to 44.7] | 1.06[16.44 to 18.17] | 1.88[13.46 to 60.34] | NA |
| Sample size | <150 | 1.68[2.72 to 7.39] | 0.37[0.20 to 0.62] | 0.64[0.27 to 1.67] | 0.41[0.17 to 1.02] | 0.41[0.20 to 0.85] | 0.67[0.30 to 1.55] | 0.37[0.04 to 4.06] |
|  | ≥150 | 0.85[0.57 to 1.28] | 0.33[0.20 to 0.53] | 0.46[0.27 to 0.76] | NA | NA | 0.30[0.18 to 0.47] | 0.49[0.09 to 2.56] |
| Center | Single | 1.09[1.49 to 1.82] | 0.42[0.25 to 0.70] | 0.61[0.37 to 1.11] | NA | 0.33[0.14 to 0.87] | 0.20[0.09 to 0.52] | 0.30[0.08 to 1.28] |
|  | Multiple | 0.79[0.42 to 1.54] | 0.33[0.17 to 0.63] | 0.44[0.20 to 0.93] | 0.25[0.11 to 0.60] | 0.40[0.14 to 0.84] | 0.33[0.18 to 0.70] | NA |
| AEs | Reported | 0.89[0.53 to 1.51] | 0.37[0.22 to 0.57] | 0.53[0.33 to 0.88] | 0.27[0.14 to 0.54] | 0.30[0.15 to 0.67] | 0.33[0.20 to 0.59] | 0.37[0.09 to 1.32] |
|  | No reported | 1.05[1.90 to 2.27] | 0.37[0.17 to 0.88] | 0.52[0.22 to 1.65] | NA | 0.52[0.09 to 3.32] | 0.40[0.18 to 0.92] | NA |
| RoB2 | ≤7 | 0.49[0.53 to 1.72] | 0.37[0.22 to 0.64] | 0.52[0.27 to 1.09] | 0.48[0.08 to 2.59] | 0.68[0.22 to 2.10] | 0.33[0.18 to 0.59] | 0.37[0.08 to 1.58] |
|  | >7 | 0.94[0.55 to 1.62] | 0.39[0.25 to 0.65] | 0.56[0.33 to 0.99] | 0.30[0.11 to 0.70] | 0.25[0.11 to 0.59] | 0.43[0.25 to 0.79] | NA |

Table S8: Network meta-regression for recurrence and progression

| Outcome | Characteristic | ADM | BCG | EPI | GEM | IFN | MMC | THP |
| --- | --- | --- | --- | --- | --- | --- | --- | --- |
| Recurrence | Year | 0.64[0.48 to 0.85] | 0.39[0.30 to 0.53] | 0.63[0.45 to 0.86] | 0.33[0.18 to 0.58] | 0.43[0.30 to 0.61] | 0.51[0.39 to 0.68] | 0.64[0.38 to 1.08] |
|  | Sample size | 0.68[0.55 to 0.85] | 0.42[0.33 to 0.54] | 0.66[0.50 to 0.89] | 0.37[0.20 to 0.61] | 0.45[0.30 to 0.63] | 0.54[0.43 to 0.68] | 0.69[0.41 to 1.14] |
|  | Quality | 0.70[0.57 to 0.87] | 0.43[0.33 to 0.54] | 0.66[0.50 to 0.89] | 0.37[0.20 to 0.61] | 0.44[0.30 to 0.63] | 0.55[0.44 to 0.68] | 0.69[0.41 to 1.15] |
| Progression | Year | 0.97[0.62 to 1.55] | 0.37[0.22 to 0.56] | 0.53[0.33 to 0.85] | 0.30[0.15 to 0.56] | 0.39[0.20 to 0.74] | 0.37[0.22 to 0.59] | 0.38[0.09 to 1.45] |
|  | Sample size | 0.90[0.64 to 0.78] | 0.30[0.20 to 0.45] | 0.45[0.30 to 0.68] | 0.25[0.14 to 0.44] | 0.30[0.15 to 0.60] | 0.33[0.22 to 0.47] | 0.33[0.09 to 1.09] |
|  | Quality | 0.94[0.66 to 1.35] | 0.33[0.25 to 0.48] | 0.51[0.33 to 0.75] | 0.27[0.15 to 0.51] | 0.37[0.20 to 0.71] | 0.37[0.25 to 0.51] | 0.38[0.10 to 1.38] |

Table S9: Ranking analysis for recurrence

|  | ADM | BCG | EPI | GEM | IFN | MMC | THP | TURBT |
| --- | --- | --- | --- | --- | --- | --- | --- | --- |
| Rank 1 | 0.0000 | 0.1359 | 0.0001 | 0.6851 | 0.1691 | 0.0002 | 0.0096 | 0.0000 |
| Rank 2 | 0.0001 | 0.5239 | 0.0016 | 0.1493 | 0.2955 | 0.0070 | 0.0226 | 0.0000 |
| Rank 3 | 0.0011 | 0.3189 | 0.0164 | 0.1058 | 0.3899 | 0.1179 | 0.0501 | 0.0000 |
| Rank 4 | 0.0139 | 0.0206 | 0.0837 | 0.0374 | 0.1051 | 0.6257 | 0.1136 | 0.0000 |
| Rank 5 | 0.1983 | 0.0008 | 0.3399 | 0.0140 | 0.0305 | 0.2127 | 0.2039 | 0.0000 |
| Rank 6 | 0.4435 | 0.0000 | 0.3318 | 0.0055 | 0.0082 | 0.0332 | 0.1774 | 0.0003 |
| Rank 7 | 0.3428 | 0.0000 | 0.2244 | 0.0027 | 0.0018 | 0.0034 | 0.3520 | 0.0729 |
| Rank 8 | 0.0003 | 0.0000 | 0.0021 | 0.0001 | 0.0000 | 0.0000 | 0.0708 | 0.9268 |
| Best rank | 6 | 2 | 5 | 1 | 3 | 4 | 7 | 8 |
| SUCRA | 0.2695 | 0.8248 | 0.3335 | 0.9180 | 0.7765 | 0.5490 | 0.3180 | 0.0105 |
| Rank | 7 | 2 | 5 | 1 | 3 | 4 | 6 | 8 |

Table S10: Ranking analysis for progression

|  | ADM | BCG | EPI | GEM | IFN | MMC | THP | TURBT |
| --- | --- | --- | --- | --- | --- | --- | --- | --- |
| Rank 1 | 0.0000 | 0.1359 | 0.0001 | 0.6851 | 0.1691 | 0.0002 | 0.0096 | 0.0000 |
| Rank 2 | 0.0001 | 0.5239 | 0.0016 | 0.1493 | 0.2955 | 0.0070 | 0.0226 | 0.0000 |
| Rank 3 | 0.0011 | 0.3189 | 0.0164 | 0.1058 | 0.3899 | 0.1179 | 0.0501 | 0.0000 |
| Rank 4 | 0.0139 | 0.0206 | 0.0837 | 0.0374 | 0.1051 | 0.6257 | 0.1136 | 0.0000 |
| Rank 5 | 0.1983 | 0.0008 | 0.3399 | 0.0140 | 0.0305 | 0.2127 | 0.2039 | 0.0000 |
| Rank 6 | 0.4435 | 0.0000 | 0.3318 | 0.0055 | 0.0082 | 0.0332 | 0.1774 | 0.0003 |
| Rank 7 | 0.3428 | 0.0000 | 0.2244 | 0.0027 | 0.0018 | 0.0034 | 0.3520 | 0.0729 |
| Rank 8 | 0.0003 | 0.0000 | 0.0021 | 0.0001 | 0.0000 | 0.0000 | 0.0708 | 0.9268 |
| Best rank | 6 | 2 | 5 | 1 | 3 | 4 | 7 | 8 |
| SUCRA | 0.0992 | 0.6931 | 0.3669 | 0.8716 | 0.6103 | 0.6674 | 0.6299 | 0.0615 |
| Rank | 7 | 2 | 6 | 1 | 5 | 3 | 4 | 8 |


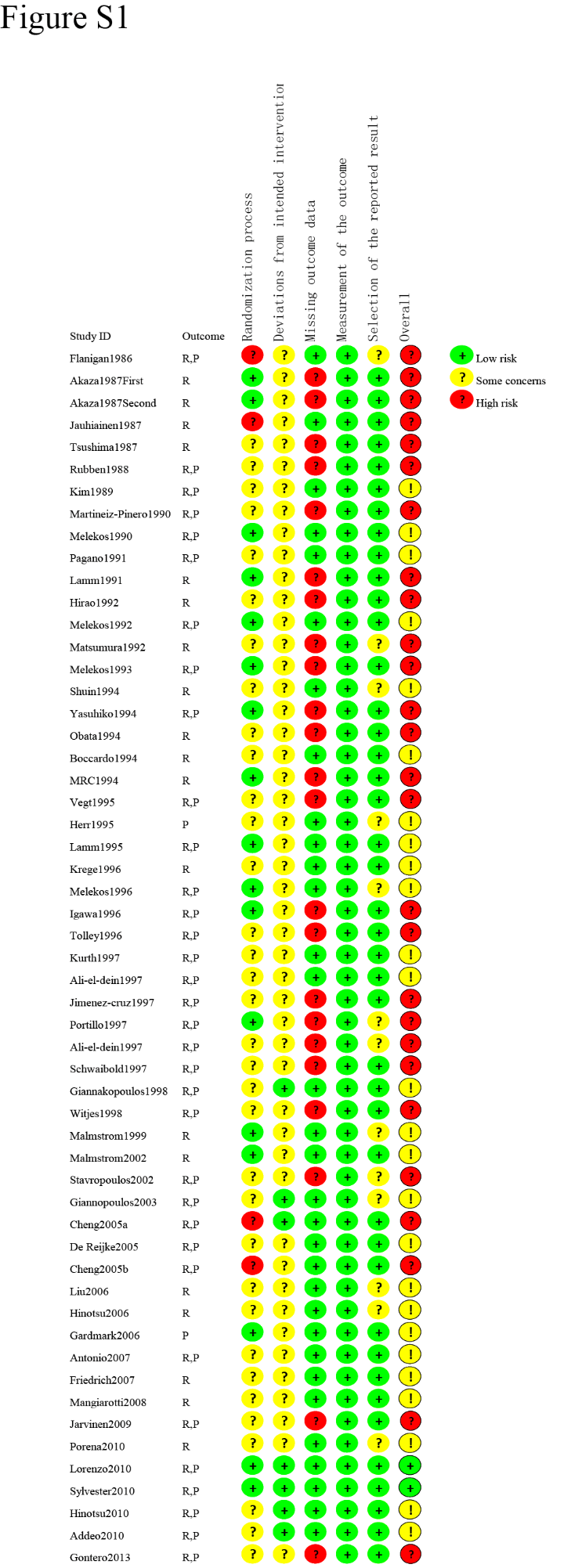


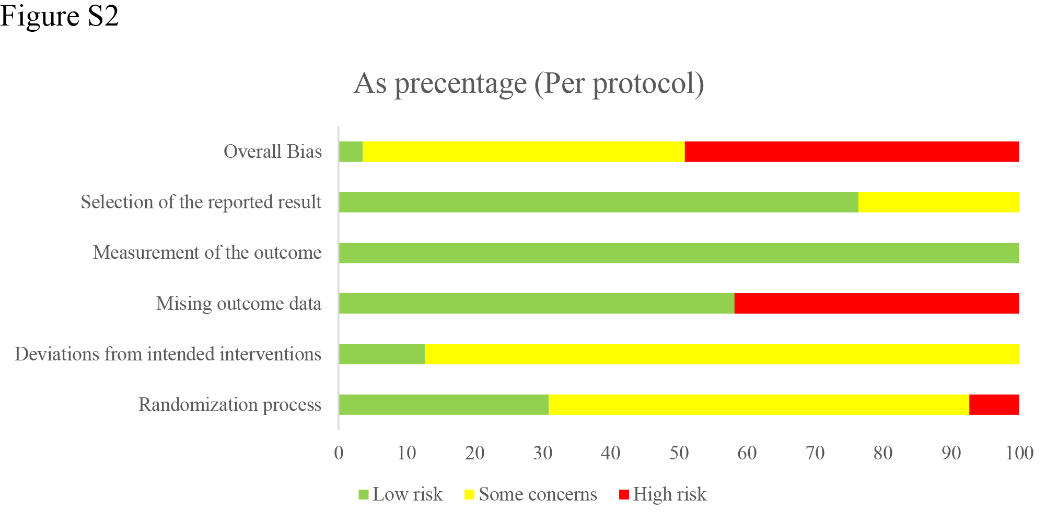


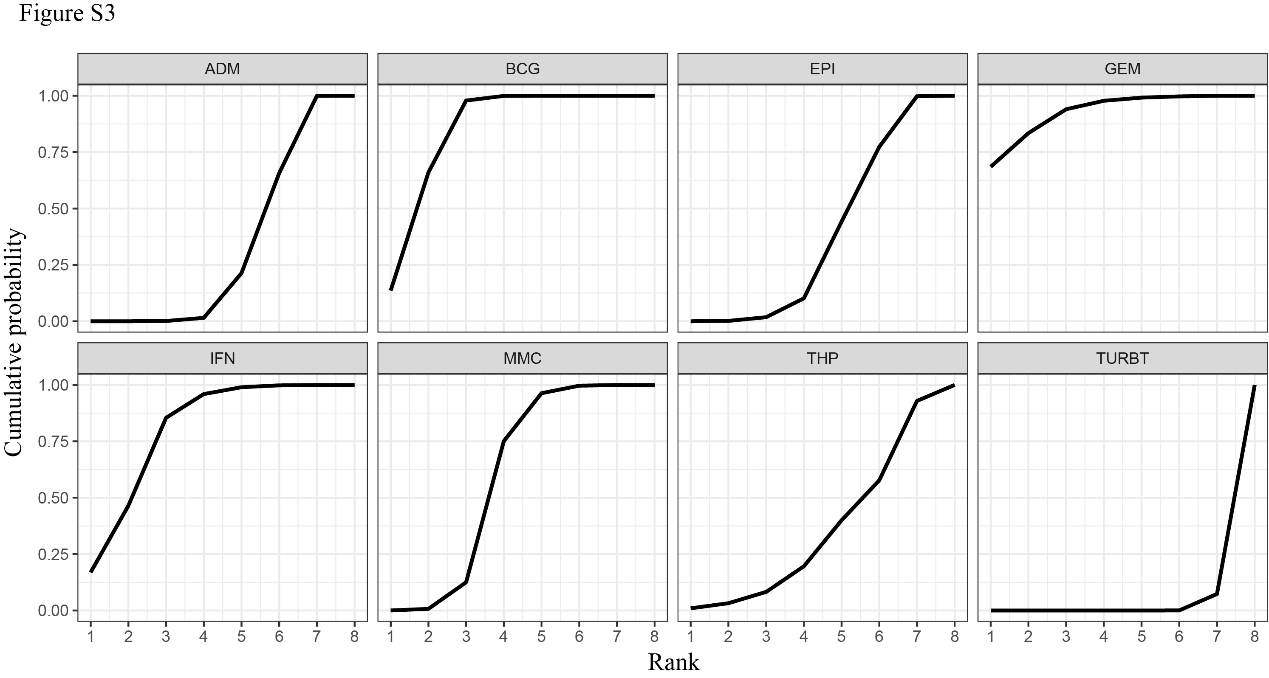


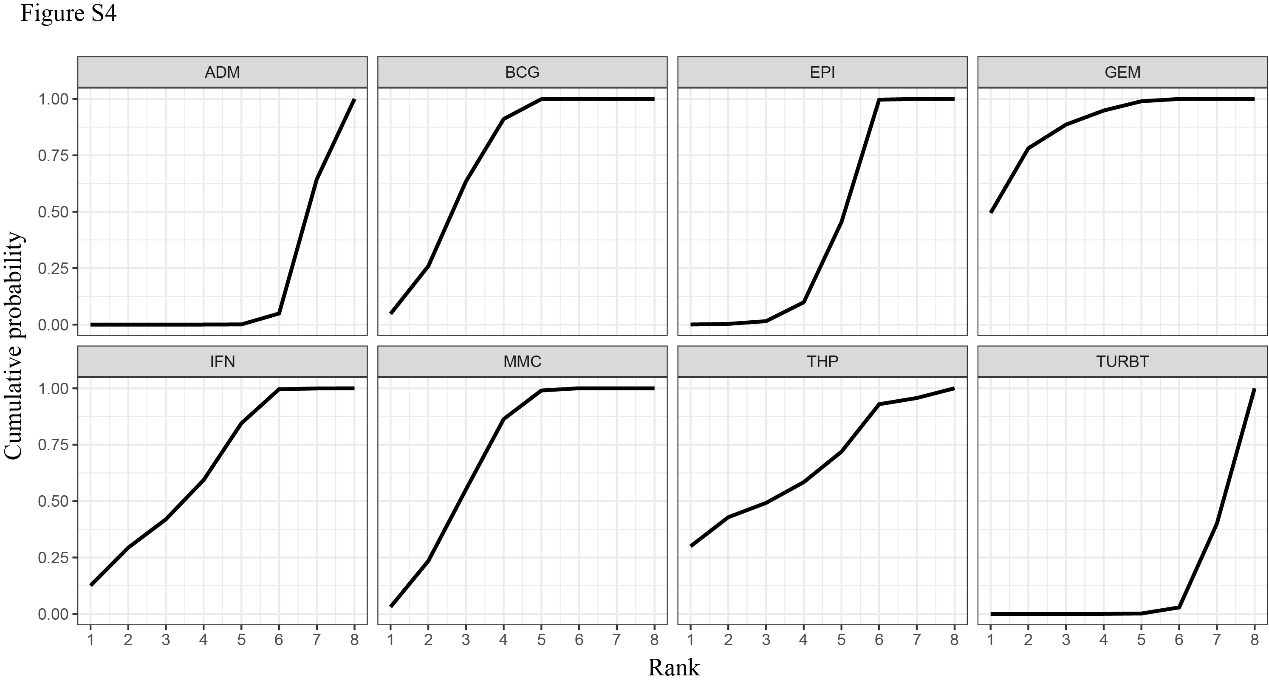

Supplement: Supplementary file 5 — File S5 [file CAM4-9-7800-s005.docx]
